# SS18-SSX drives CREB activation in synovial sarcoma

**DOI:** 10.1007/s13402-022-00673-w

**Published:** 2022-05-12

**Authors:** Magdalene Cyra, Miriam Schulte, Ruth Berthold, Lorena Heinst, Esther-Pia Jansen, Inga Grünewald, Sandra Elges, Olle Larsson, Christoph Schliemann, Konrad Steinestel, Susanne Hafner, Thomas Simmet, Eva Wardelmann, Sareetha Kailayangiri, Claudia Rossig, Ilka Isfort, Marcel Trautmann, Wolfgang Hartmann

**Affiliations:** 1 Division of Translational Pathology, Gerhard-Domagk-Institute of Pathology, Münster University Hospital, Münster, Germany; 2 Gerhard-Domagk-Institute of Pathology, Münster University Hospital, Münster, Germany; 3 Departments of Oncology and Pathology, The Karolinska Institute, Stockholm, Sweden; 4 Department of Medicine A, Hematology, Oncology and Respiratory Medicine, Münster University Hospital, Münster, Germany; 5 Institute of Pathology and Molecular Pathology, Bundeswehrkrankenhaus Ulm, Ulm, Germany; 6 Institute of Pharmacology of Natural Products and Clinical Pharmacology, Ulm University, Ulm, Germany; 7 Department of Pediatric Hematology and Oncology, University Children’s Hospital Münster, Münster, Germany

**Keywords:** Synovial sarcoma, SS18-SSX, CREB, 666-15, BMS-754807

## Abstract

**Purpose:**

Synovial sarcoma (SySa) is a rare soft tissue tumor characterized by a reciprocal t(X;18) translocation. The chimeric SS18-SSX fusion protein represents the major driver of the disease, acting as aberrant transcriptional dysregulator. Oncogenic mechanisms whereby SS18-SSX mediates sarcomagenesis are incompletely understood, and strategies to selectively target SySa cells remain elusive. Based on results of Phospho-Kinase screening arrays, we here investigate the functional and therapeutic relevance of the transcription factor CREB in SySa tumorigenesis.

**Methods:**

Immunohistochemistry of phosphorylated CREB and its downstream targets (Rb, Cyclin D1, PCNA, Bcl-xL and Bcl-2) was performed in a large cohort of SySa. Functional aspects of CREB activity, including SS18-SSX driven circuits involved in CREB activation, were analyzed in vitro employing five SySa cell lines and a mesenchymal stem cell model. CREB mediated transcriptional activity was modulated by RNAi-mediated knockdown and small molecule inhibitors (666-15, KG-501, NASTRp and Ro 31-8220). Anti-proliferative effects of the CREB inhibitor 666-15 were tested in SySa avian chorioallantoic membrane and murine xenograft models in vivo.

**Results:**

We show that CREB is phosphorylated and activated in SySa, accompanied by downstream target expression. Human mesenchymal stem cells engineered to express SS18-SSX promote CREB expression and phosphorylation. Conversely, RNAi-mediated knockdown of *SS18-SSX* impairs CREB phosphorylation in SySa cells. Inhibition of CREB activity reduces downstream target expression, accompanied by suppression of SySa cell proliferation and induction of apoptosis *in* *vitro* and in vivo*.*

**Conclusion:**

In conclusion, our data underline an essential role of CREB in SySa tumorigenesis and provides evidence for molecular targeted therapies.

**Supplementary Information:**

The online version contains supplementary material available at 10.1007/s13402-022-00673-w.

## Introduction

Synovial sarcoma (SySa) is a rare soft tissue tumor comprising approximately 2% of all malignant soft tissue neoplasms, occurring predominantly in adolescents and young adults [1–3]. Wide surgical resection, radiotherapy and chemotherapy represent well-established therapeutic options, but the prognosis in the metastatic situation is poor. Specific targeted molecular therapeutic approaches are currently not available [4, 5].

Genetically, SySa are characterized by a pathognomonic reciprocal t(X;18) translocation, leading to the fusion of *SS18* to one of the homologous *SSX* genes (most frequently *SSX1* or *SSX2*, in rare cases *SSX4*), generating a chimeric SS18-SSX fusion protein [6–8]. Though the pathognomonic SS18-SSX fusion protein is known to play a crucial role in SySa tumorigenesis, its exact biological function still remains to be determined. It is known that the SS18-SSX proteins interact with the chromatin-remodeling SWI/SNF and Polycomb complexes [9, 10], playing a role as an aberrant transcription dysregulator which is involved in the dysregulation of oncogenic signaling pathways, including the Insulin-like growth factor (IGF)/PI3K/Akt, WNT and Hippo pathways [11–16].

The cAMP response element-binding protein CREB is a ubiquitously expressed transcription factor that binds to the conserved palindromic CRE (cAMP regulated enhancer) element sequence (5’-TGACGTCA-3’) located in regulatory promotor regions of up to 4000 genes, as identified in a genome-wide analysis, suggesting that CREB may play an important role in orchestrating several gene sets [17–19]. It has been found that *Creb* knockout in mice results in developmental impairment associated with early perinatal death, emphasizing its crucial role in development and cell physiology [20]. The CREB protein contains highly conserved bZIP-domains involved in direct DNA interactions [21], a KID (kinase inducible)-domain [22] necessary for interaction with coactivators, and a Q2-domain (glutamine-rich constitutive activator) cooperating with the KID-domain to stimulate target gene expression [23]. CREB activation, e.g. through an increase of intracellular cAMP levels due to extracellular stimuli, is realized through phosphorylation of serine 133 (S133) by different kinases including PKA, Akt (PKB) and p90RSK [24–30]. Activated CREB forms homo- or heterodimers with other transcription factors to subsequently bind CRE-elements in promoter regions, thereby inducing gene transcription in cooperation with the histone acetyl transferase CREB binding protein (CBP) [17, 27, 31, 32]. The interaction of CREB and CBP is realized through binding of the KID-domain of CREB to the KIX (KID-interacting) domain of CBP, making it a possible structural target in the inhibition of CREB activity [24]. While pharmacological inhibition of the CREB-CRE interaction is challenging, CREB-dependent gene transcription may efficiently be modulated through inhibition of the interaction of CREB and CBP or the antagonization of upstream kinases.

An essential role of CREB is well documented in different epithelial cancers and acute myeloid leukemia, with CREB overactivation being associated with a poor prognosis [33–36]. As yet, the function of CREB in SySa has not been evaluated, but pathogenic translocations between *EWSR1* and *CREB* or its homologue *ATF1* in clear cell sarcoma, another fusion-driven mesenchymal neoplasia, point to a major functional role in mesenchymal tumorigenesis [37, 38]. The current preclinical study was performed to explore the prevalence and functional relevance of CREB signals in a large set of human SySa specimens, including its molecular dependence on the pathognomonic SS18-SSX fusion protein, and to test molecularly targeted approaches employing small molecule CREB inhibitors in vitro and in vivo*.*

## Materials and methods

### Tumor specimens and tissue microarray (TMA) construction

Formalin-fixed paraffin-embedded (FFPE) SySa tumor specimens were selected from the archives of the Gerhard-Domagk-Institute of Pathology (Münster University Hospital, Germany). All diagnoses were reviewed by two experienced pathologists (EW, WH) based on clinical information, morphological criteria, and *SS18* break-apart fluorescence in situ hybridization (FISH) or reverse transcriptional PCR (RT-PCR) analysis according to the current WHO classification of tumours of Soft Tissue and Bone [2]. Tissue microarrays (TMA) were constructed with two representative 1 mm cores included from each tumor sample selected by experienced pathologists (EW, WH) in order to represent potential heterogeneity. Occasionally occurring necrobiotic areas and their neighborhood were excluded from TMA sampling to avoid the detection of secondary (e.g. hypoxia-induced) alterations. In total, 65 SySa tumor specimens were included in the cohort (30 women, 35 men; median age at diagnosis: 45 years, range: 8–78 years of age). The median tumor size was 5 cm (range: 0.3–20 cm). Clinicopathological characteristics of the cohort are listed in Table 1. Analysis of the cohort of mesenchymal tumors for research purposes was approved by the Ethics Review Board of the University of Münster (2015–548-f-S). All experiments were conformed to the principles set out in the World Medical Association Declaration of Helsinki and the United States Department of Health and Human Services Belmont Report.

**Table 1:** Clinicopathological characteristics of SySa patients (n = 65)

Age (years)		
*Mean (*± *SD)*	41	(± 17)
*Median (range)*	45	(8–78)
< *41*	28	(43.1%)
≥ *41*	37	(56.9%)
Type		
*Primary tumor*	43	(66.2%)
*Metastasis*	9	(13.8%)
*Recurrence*	7	(10.8%)
*ND*	6	(9.2%)
Morphology		
*Monophasic*	48	(73.4%)
*Biphasic*	14	(21.5%)
*Poorly differentiated*	3	(4.6%)
Size (cm)		
*Mean (*± *SD)*	7	(± 5)
*Median (range)*	5	(0.3–20)
< *7*	32	(49.2%)
≥ *7*	18	(27.7%)
*ND*	15	(23.1%)
Sex		
*Female*	30	(46.2%)
*Male*	35	(53.8%)
FISH		
*SS18* (break-apart) positive	52	(80%)
ND	13	(20%)
t(X;18) translocation type		
*SS18-SSX1*	28	(43.1%)
*SS18-SSX2*	20	(30.8%)
ND	17	(26.1%)
Grading (FNCLCC)		
G2	16	(24.6%)
G3	24	(36.9%)
ND	25	(38.5%)

*SD* standard deviation; *ND* not determined; *FISH* fluorescence in situ hybridization

### Immunohistochemistry (IHC)

The following primary antibodies were applied: anti-phospho (p)-CREB (S133, monoclonal rabbit, 87G3; Cell Signaling #9198), anti-phospho (p)-Akt (S473, monoclonal rabbit, D9E; Cell Signaling #4060), anti-Rb (monoclonal mouse, G3-245; BD Biosciences #554,136), anti-Cyclin D1 (monoclonal rabbit, SP4-R; Ventana #790–4508), anti-PCNA (monoclonal rabbit, D3H8P; Cell Signaling #13,110), anti-Bcl-xL (monoclonal rabbit, 54H6; Cell Signaling #2764S), anti-Bcl-2 (monoclonal mouse, 124; Ventana #790–4464), anti-cleaved Caspase 3 (N175, polyclonal rabbit; Cell Signaling #9661) and anti-phospho (p)-Histone H3 (S10, rabbit; Cell Signaling #9701). IHC staining was performed using a BenchMark ULTRA Autostainer (VENTANA/Roche) on 3 μm tissue sections. In brief, the staining procedure included: (i) heat-induced epitope retrieval pre-treatment using Tris–Borate-EDTA buffer (pH 8.4 or pH 6.0; 95–100 °C, 32–72 min) followed by (ii) incubation with respective primary antibodies for 16–120 min and (iii) employment of an OptiView DAB IHC Detection Kit (VENTANA/Roche). Positive and negative control stainings using an appropriate IgG subtype (DCS) were included. Immunoreactivity was assessed using a semi-quantitative score (0, negative; 1, weak; 2, moderate; and 3, strong) defining the staining intensity in the positive controls (breast cancer, NST) as strong. Only TMA tissue cores with at least moderate staining (semi-quantitative score ≥ 2 and ≥ 20% stained cells, except for Rb: ≥ 1 and ≥ 10%) were considered positive for the purposes of the study. The IHC readers were blinded to outcome data and the cut-offs were pre-specified without prior analyses of the clinical course.

### Cell culture and cell lines

The human SySa cell lines HS-SY-II (expressing SS18-SSX1), FUJI, 1273/99, CME-1 and SYO-I (all expressing SS18-SSX2) were cultured as described previously [13]. For the purpose of cell line authentication, expression of the pathognomonic *SS18-SSX* fusion gene was confirmed by RT-PCR and Sanger sequencing using specific primers for the t(X;18) translocation subtypes. The human mesenchymal stem cell line SCP-1 was kindly provided by Dr. Attila Aszodi, Munich, Germany. The lung adenocarcinoma cell line A549 was obtained from the American Type Culture Collection (ATCC). All cells were grown under standard incubation conditions (37 °C, humidified atmosphere, 5% CO_2_) and maintained in Dulbecco's Modified Eagle medium (DMEM; HS-SY-II, SYO-I and A549), Roswell Park Memorial Institute 1640 medium (RPMI-1640; FUJI, CME-1), F-12 (1273/99) or Minimum Essential Medium (MEM; SCP-1), supplemented with 10–15% fetal bovine serum (FBS; Life Technologies). Mycoplasma testing was performed quarterly by standardized PCR, and cells were passaged for a maximum of 20–30 culturing cycles between thawing and the experiments. To study the biological effects of treatment with small molecule inhibitors, SySa cells were grown in medium supplemented with 2% FBS. Cell lysis, protein extraction and immunoblotting were performed 6–72 h after treatment as previously described [39].

### Phospho-kinase array

HS-SY-II cells were grown in medium supplemented with 10% FBS for 48 h until a cell density of 70% was reached. Next, protein extraction and phospho-kinase immunoblotting combining spotted antibodies for 46 kinase phosphorylation sites were performed as indicated by the manufacturer (Human Phospho-Kinase Array Kit, #ARY003, R&D Systems). Immunoblot development was performed using a chemiluminescence detection kit (ECL Amersham) and a Molecular Imager ChemiDoc system (Image Lab Software; Bio-Rad). Densitometric analysis was conducted using ImageJ software (http://rsb.info.nih.gov/ij).

### Transient *SS18-SSX* fusion gene expression

The generation of a *SS18-SSX2* expression plasmid has been described previously [39]. Human SCP-1 mesenchymal stem cells were grown in 6-well cell culture plates under standard culture conditions and transfected with *SS18-SSX2* pDNA or the pT-REx/GW-30/lacZ plasmids expressing β-galactosidase (*LacZ*; Life Technologies) using Viromer Red (Lipocalyx). Next, the cells were lysed, total protein extracts were isolated and SS18-SSX2 expression was confirmed by immunoblotting 24 h after transfection. To confirm suitability of *SS18-SSX2* expressing SCP-1 cells as a model system for SySa, mRNA expression of *ATF3*, *EGR1* and *CDKN2A* was assessed, showing downregulation of these transcripts by *SS18-SSX2*, as to be expected from previous studies in SySa (Supplementary Fig. S2) [10].

### Immunoblotting

The following primary antibodies were used: anti-Akt (monoclonal rabbit, C67E7, #4691), anti-phospho (p)-Akt (S473, monoclonal rabbit, D9E, #4060), anti-Bcl-xL (monoclonal rabbit, 54H6, #2764), anti-Bcl-2 (polyclonal rabbit, #2876), anti-Cyclin D1 (monoclonal rabbit, 92G2, #2978), anti-CREB (monoclonal rabbit, 48H2, #9197), anti-p-CREB (S133, monoclonal rabbit, 87G3, #9198), anti-GAPDH (monoclonal rabbit, D16H11, #5174), anti-IGF-IR (monoclonal rabbit, D23H3, #9750 and monoclonal rabbit, D4O6W, #14,534), anti-phospho (p)-IGF-IR (Y1135/1136, monoclonal rabbit, 19H7, #3024), anti-PCNA (monoclonal rabbit, D3H8P, #13,110) and anti-Rb (polyclonal rabbit, #9313) (all purchased from Cell Signaling Technology). The SS18-SSX fusion protein was visualized using an anti-SS18/SYT antibody (#8819 Santa Cruz Biotechnology) detecting the N-terminus of SS18 (which is retained in the SS18-SSX fusion oncoprotein). Secondary antibody labeling (Bio-Rad) and immunoblot development were performed using an enhanced chemiluminescence detection kit (SignalFire ECL Reagent; Cell Signaling Technology) and a Molecular Imager ChemiDoc system (Image Lab Software; Bio-Rad), as described previously [14, 40].

### RNA interference (RNAi)

A set of pre-validated Stealth siRNAs for *CREB*, #2 = HSS102264, #3 = HSS175171; for *IGF-IR*: #4 = siRNA ID #110,754, #5 = siRNA ID #74 and a non-targeting negative control siRNA (BLOCK-iT Alexa Fluor Red Fluorescent Control) were purchased from Life Technologies. To target the *SS18-SSX* fusion transcript, a set of published and pre-validated duplex oligos was employed (#1 sense: 5′-AAC CAA CUA CCU CUG AGA AGA-3′; antisense: 5′-UCU UCU CAG AGG UAG UUG GUU-3′, #2 sense: 5′-CAA GAA GCC AGC AGA GGA ATT-3′; antisense: 5′-UUC CUC UGC UGG CUU CUU GTT-3′ and #3 sense: 5′-AUA UGA CCA GAU CAU GCC CAA GAU U-3′; antisense: 5′-UCU UGG GCA UGA UCU GGU CAU AUU U-3′) [41, 42]. For RNAi experiments, HS-SY-II, CME-1 and 1273/99 cells were cultured in 25 cm^2^ cell culture flasks (medium supplemented with 2% FBS) and transfected with indicated siRNAs (25 pmol; cell density of 50%) using Lipofectamine RNAiMAX (Life Technologies). After incubation for 48–72 h, siRNA-transfected cells were lysed, proteins were extracted, and knockdown efficiency was documented by immunoblotting.

### Quantitative real time PCR (qRT-PCR)

RNA was extracted using a RNeasy Plus Mini Kit (QIAGEN) and converted into complementary DNA (cDNA) using a ProtoScript II First Strand cDNA Synthesis Kit (New England Biolabs) according to the manufacturer’s instructions. Quantitative PCR (qPCR) was performed using a StepOnePlus Real-Time PCR System (Thermo Fisher Scientific), applying a Power SYBR Green PCR Master Mix (Thermo Fisher Scientific). The following primer sets were used: *ATF3:* for 5′-ATCGTCCCCTGCCTGTCCCC-3′ and rev 5′-CCCGAGGGGTCTGTCGCTGA-3′. *BCL2:* for 5′-CGGGAGATGTCGCCCCTGGT-3′ and rev 5′-GCATGCTGGGGCCGTACAGT-3′. *CCND1:* for 5′-AACTACCTGGACCGCTTCCT-3′ and rev 5′-CCACTTGAGCTTGTTCACCA-3′.*CDKN2A:* for 5′-ACGAGGCACCTTGGAAACAGGTAT-3′ and rev 5′-AGAACGTGGCTTTAAGGTCTGGGA-3′. *CREB1:* for 5′-CCAGCAGAGTGGAGATGCAG-3′ and rev 5′-GGGCTAATGTGGCAATCTGTG-3′. *EGR1:* for 5′-GGTCAGTGGCCTAGTGAGC-3′ and rev 5′-GTGCCGCTGAGTAAATGGGA-3′. *GAPDH* (as reference housekeeper gene): for 5′-CTCTGCTCCTCCTGTTCGAC-3′ and rev 5′-TTAAAAGCAGCCCTGGTGAC-3′. *SS18-SSX1:* for 5′-CCAGCAGAGGCCTTATGGAT-3′ and rev 5′-GGTGCAGTTGTTTCCCATCG-3′. *SS18-SSX2:* for 5′-CCAGCAGAGGCCTTATGGAT-3′ and rev 5′- GGCACAGCTCTTTCCCATCA-3′. StepOne software (Thermo Fisher Scientific) was used for data interpretation. Relative mRNA expression fold changes of gene expression were calculated using the 2 − ΔΔCt method.

### Compounds

The following compounds were dissolved in dimethyl sulfoxide (DMSO; Sigma-Aldrich): 666-15 (inhibitor of CREB-mediated gene transcription; C_33_H_30_ClN_3_O_5_.HCl; CAS#: 1,433,286-70-4; TOCRIS #5661); KG-501 (Naphthol AS-E phosphate) (binds KIX-domain of CBP and prevents interaction between CREB and CBP; C_17_H_13_ClNO_5_P; CAS#: 18,228-17-6; Sigma-Aldrich #70,485); NASTRp (Naphthol AS-TR phosphate disodium salt) (modified from Naphthol analog KG-501, binds KIX-domain of CBP and disrupts association between KIX and KID domain; C_18_H_13_ClNNa_2_O_5_P; CAS#: 4264-93-1, Sigma-Aldrich #N6125); Ro 31-8220 (Bisindolylmaleimide IX) (pan-PKC inhibitor, C_25_H_23_N_5_O_2_S·CH_4_O_3_S; CAS#: 138,489-18-6; Selleckchem #S7207); and BMS-754807 (IGF-IR/IR ATP antagonist; C_23_H_24_FN_9_O; CAS#: 1,001,350-96-4; Biomol #LKT-B5072.1). The final DMSO concentration did not exceed 0.2% (v/v) for all in vitro and in vivo applications.

### Insulin-like growth factor-II (IGF-II) stimulation

Stimulation of SySa cells with 200 ng/ml IGF-II protein (human recombinant (rHu)IGF-2; Biomol #ARG70131.100) was performed for 5 min after starving the cells for 6 h in FBS-depleted medium. Stimulated cells were lysed, total proteins were isolated and analysis was performed by immunoblotting as described previously [39].

### Cell viability assay (MTT)

For cell viability assessment a MTT cell proliferation kit (Roche) was used. In brief, HS-SY-II (15 × 10^3^), CME-1 (6 × 10^3^), SYO-I (8 × 10^3^), FUJI (10 × 10^3^), 1273/99 (7 × 10^3^) and A549 (2.5 × 10^3^) cells were seeded in 96-well cell culture plates (100 µl medium supplemented with 2% FBS) and exposed to increasing small molecule compound concentrations: 666-15 [43, 44] (0.13-2.5 µmol/L), KG-501 [45] (0.63-5 µmol/L), NASTRp [46] (0.63-2.5 µmol/L) and Ro 31-8220 [47] (0.13-2 µmol/L) for 72 h. An appropriate DMSO vehicle control was included. At least three independent experiments were performed (each in quintuplicates) and results were calculated as mean ± SEM.

### ApoTox-Glo Triplex cell viability, cytotoxicity and apoptosis assay

The ApoTox-Glo Triplex assay (Promega) was applied to assess cell viability, cytotoxicity and apoptosis. CME-1 cells were cultured in FBS-reduced medium (RPMI + 2% FBS) and incubated with decreasing concentrations of the CREB-inhibitor 666-15 (0.09-0.18 µmol/L) or an appropriate DMSO vehicle control for 20 h. Fluorogenic peptide substrate GF-AFC was used to evaluate cell viability, and Caspase 3/7 activation was employed as an indicator of apoptosis. Fluorescence signals were analysed in quintuplicate using a GloMax Microplate Reader (Promega).

### Chicken chorioallantoic membrane (CAM) studies

SYO-I cells (1.5 × 10^6^ cells/egg; dissolved in medium/Matrigel 1:1, v/v) were deposited within 5 mm silicon rings on the surface of chicken chorioallantoic membranes seven days post fertilization and incubated at 37 °C with 60% relative humidity as previously described [48]. For drug treatment, 0.5 µmol/L 666-15 or vehicle CTRL (0.2% DMSO in NaCl 0.9%) were applied topically on day 8, 9 and 10. Three days after treatment initiation, xenografts were explanted.

### SySa cell xenograft studies

SYO-I cells (5 × 10^6^ / 100 µl PBS) were injected subcutaneously in the lower flank of NOD scid gamma (NSG) mice. Once tumors reached a volume of approximately 100 mm^3^ (14 days after cell injection), tumor-bearing mice were homogeneously distributed across three treatment groups: (i) 10 mg/kg 666-15, (ii) 20 mg/kg 666-15 or (iii) DMSO vehicle control. Intraperitoneal administration was performed every other day. Tumor volumes (mm^3^) were calculated according to the formula: length (mm) x width^2^ (mm) x π/6 [49]. For all animal studies, permission was obtained from local authorities in accordance to EU legislation, and all in vivo experiments were performed in accordance with EU Directive 2010/63/EU for animal experiments.

### Statistical analysis

Two-group comparisons were analysed by unpaired, two-tailed Student *t* test (GraphPad Software). Results of MTT/ApoTox-Glo Triplex assays and flow cytometric analyses are presented as mean ± SEM (standard error of the mean) from *n* independent experiments (n ≥ 3). Statistical differences were considered significant at *p* < 0.05 (*), *p* < 0.01 (**) and *p* < 0.001(***). The concentrations of pharmacological inhibitors (666-15, KG-501, NASTRp and PKC-inhibitor Ro31-8220) required for 50% growth inhibition (IC_50_ value), were calculated by non-linear regression analysis.

## Results

### Identification of phosphorylated CREB and its transcriptional targets in human SySa specimens and cell lines

**Fig. 1:**
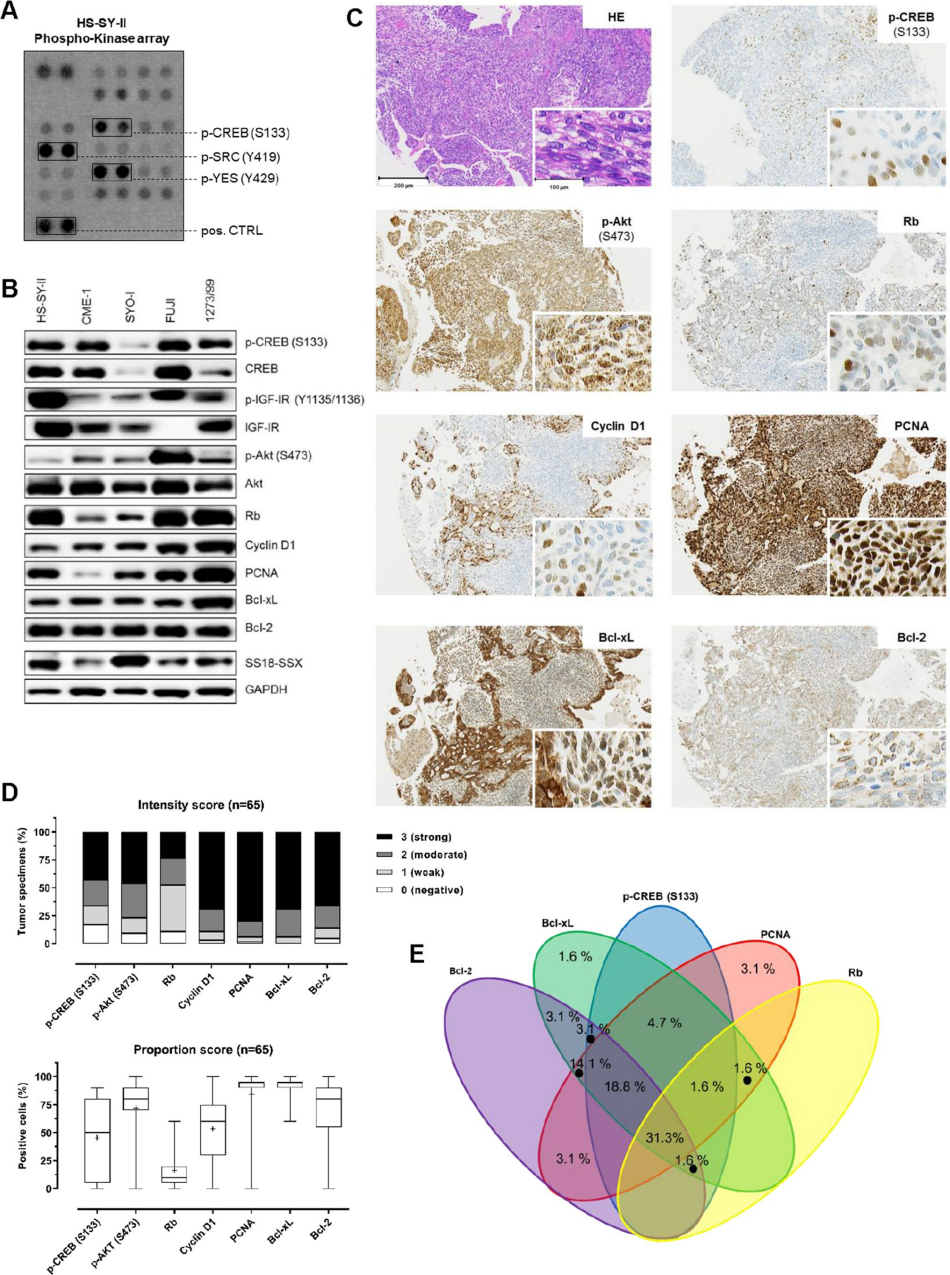
Activation patterns of CREB in synovial sarcoma tissue specimens (n = 65) and cell lines. **A** Phospho-kinase array analysis of HS-SY-II cells identifies activating CREB S133 phosphorylation to be prominent in SySa cells along with the known activation of SRC family kinases [39]. **B** Immunoblotting showing elevated expression and phosphorylation levels of CREB (S133) as well as downstream targets (Rb, Cyclin D1, PCNA, Bcl-xl and Bcl-2) and Akt (S473) in total protein extracts of five different SySa cell lines. **C** Immunohistochemical staining showing elevated phosphorylation levels of CREB (S133) as well as significant expression of Cyclin D1, Bcl-2, PCNA, Bcl-xL, Rb and p-Akt (S473) in a representative case of SySa (original magnification: 20x, inset 40x). **D** Summary of the immunohistochemical analyses of human SySa tissue specimens documented as intensity and proportion scores. IHC positivity was defined as semi-quantitative intensity score ≥ 2 and proportion ≥ 20% stained cells, except for Rb: ≥ 1 (intensity) and ≥ 10% (proportion). In the graph on the proportion score whiskers range from minimum to maximum, 25th percentile, median and 75th percentile; + represents the mean of positive cells. **E** Diagram on the concordance of p-CREB (S133) positivity and CREB target expression

Among 46 kinases phosphorylation sites screened in a human Phospho-Kinase array, CREB was detected to be strongly phosphorylated at S133 (Fig. 1A, Supplementary Fig. S1) in HS-SY-II cells. In total protein extracts of four additional SySa cell lines (CME-1, SYO-I, FUJI and 1273/99) significant protein expression of CREB and its downstream targets Rb, Cyclin D1, PCNA, Bcl-xL and Bcl-2, as well as the potential CREB regulators IGF-IR and Akt (including the phosphorylated, i.e. activated forms p-CREB S133, p-Akt S473 and p-IGF-IR Y1135/1136 (Fig. 1B) were observed.

In a set of 65 SySa specimens, expression of phosphorylated CREB (S133), the CREB downstream targets Rb, Cyclin D1, PCNA, Bcl-xL and Bcl-2, as well as the CREB upstream regulator phosphorylated Akt (S473) was examined by immunohistochemistry (Fig. 1C). Scoring was performed with regard to the staining intensity (intensity score) and the proportion of positive cells (proportion score) (Fig. 1D). Overall, 60.0% (39/65) of SySa cases were found to be positive for p-CREB (S133) (PS ≥ 20% and IS ≥ 2). Phosphorylation of CREB (S133) did not correlate with patients’ age, gender, SS18-SSX translocation subtype and/or tumor size. Immunohistochemical positivity for CREB downstream targets was as follows: Cyclin D1 (54/65 cases; 83.1%), PCNA (59/65 cases; 90.8%), Bcl-xL (61/65 cases; 93.8%) and Bcl-2 (56/65 cases; 86.1%). Rb was positive in 47.7% (31/65) of SySa cases. Expectedly, no complete concordance of p-CREB (S133) expression and immunohistochemical positivity for the downstream targets (Fig. 1E) was observed. S473 phosphorylation of Akt, known to act as an upstream regulator of CREB, was detected in 76.9% (50/65) of SySa cases. Concordance of p-Akt (S473) expression and immunohistochemical positivity for p-CREB (S133) was observed in 72.0% (36/50) of SySa specimens. 3/15 (20%) SySa cases stained positive for p-CREB (S133) without being positive for p-Akt (S473). Overall, 16.9% (11/65) of the SySa cases showed neither p-Akt (S473) nor p-CREB (S133) staining positivity.

### CREB S133 phosphorylation and CREB downstream target expression is dependent on SS18-SSX and involves an IGF-II/IGF-IR signaling loop

**Fig. 2:**
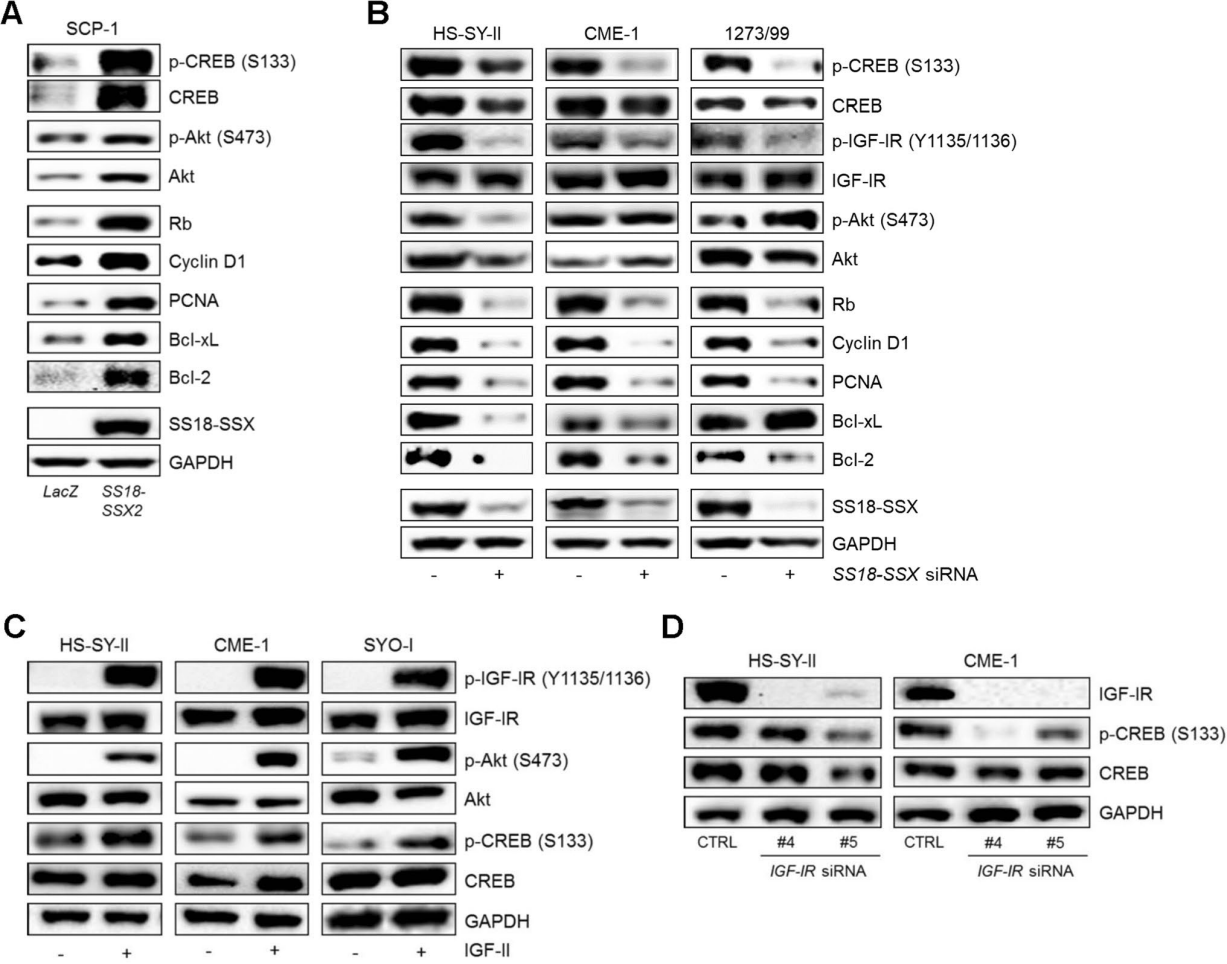
CREB expression and activation depends on SS18-SSX involving an IGF-II/IGF-IR signaling loop. **A** Transient expression of *SS18-SSX2* in SCP-1 mesenchymal stem cells induces expression and phosphorylation of CREB (S133) and phosphorylation of Akt (S473), as well as expression of the CREB downstream targets Rb, Cyclin D1, PCNA, Bcl-xl and Bcl-2 (*LacZ*, control). **B** RNAi-mediated knockdown of the SS18-SSX fusion protein is associated with a reduction in CREB (S133) and IGF-IR (Y1135/1136) phosphorylation levels and a reduced expression of CREB downstream targets (Rb, Cyclin D1, PCNA, Bcl-xl and Bcl-2) in three different SySa cell lines. **C** Serum-starved HS-SY-II, CME-1 and SYO-I cells treated with human recombinant IGF-II (200 ng/ml, 5 min) show strong activation of CREB (S133) along with IGF-IR (Y1135/1136) and Akt (S473). **D** RNAi-mediated *IGF-IR* knockdown leads to reduced CREB phosphorylation at S133

We found that transient expression of *SS18-SSX* in SCP-1 mesenchymal stem cells led to increased CREB protein and CREB S133 phosphorylation levels. Elevated protein levels of the CREB downstream targets Rb, Cyclin D1, PCNA, Bcl-xL and Bcl-2, as well as CREB´s potential “upstream” regulator phosphorylated Akt S473 (Fig. 2A) were observed. To evaluate whether CREB phosphorylation and the expression of its downstream targets are dependent on the presence of the chimeric SS18-SSX fusion protein in SySa cells, RNAi-mediated *SS18-SSX* loss-of-function analyses were performed in three SySa cell lines (HS-SY-II, CME-1 and 1273/99). Knockdown of *SS18-SSX* reduced the phosphorylation levels of CREB (S133) and (slightly) CREB expression as well as the expression levels of Rb, Cyclin D1, PCNA, Bcl-xL and Bcl-2 (Fig. 2B).

To assess whether CREB activation is dependent on IGF-IR signaling (known to play a major role in SySa), three SySa cell lines, HS-SY-II, CME-1 and SYO-I, were stimulated with IGF-II which resulted in enhanced CREB phosphorylation (S133) and, as expected, phosphorylation of IGF-IR (Y1135/1136) and Akt (S473) (Fig. 2C). To further investigate this dependency, effects of RNAi-mediated *IGF-IR* knockdown on CREB activation were tested. In HS-SY-II and CME-1 cells, efficient knockdown of *IGF-IR* with two different siRNAs led to reduced phosphorylation levels of CREB (S133) and decreased target gene expression (Rb, Cyclin D1 and Bcl-xL protein levels) in both cell lines (Fig. 2D, Supplementary Fig. S3), supporting the concept that IGF-IR signaling is involved in the activation of CREB in SySa. Comparable results were obtained by pharmacological targeting of IGF-IR with BMS-754807 (Supplementary Fig. S4A).

### Inhibition of CREB by RNAi or small molecule inhibitors impairs SySa cell viability in vitro

**Fig. 3:**
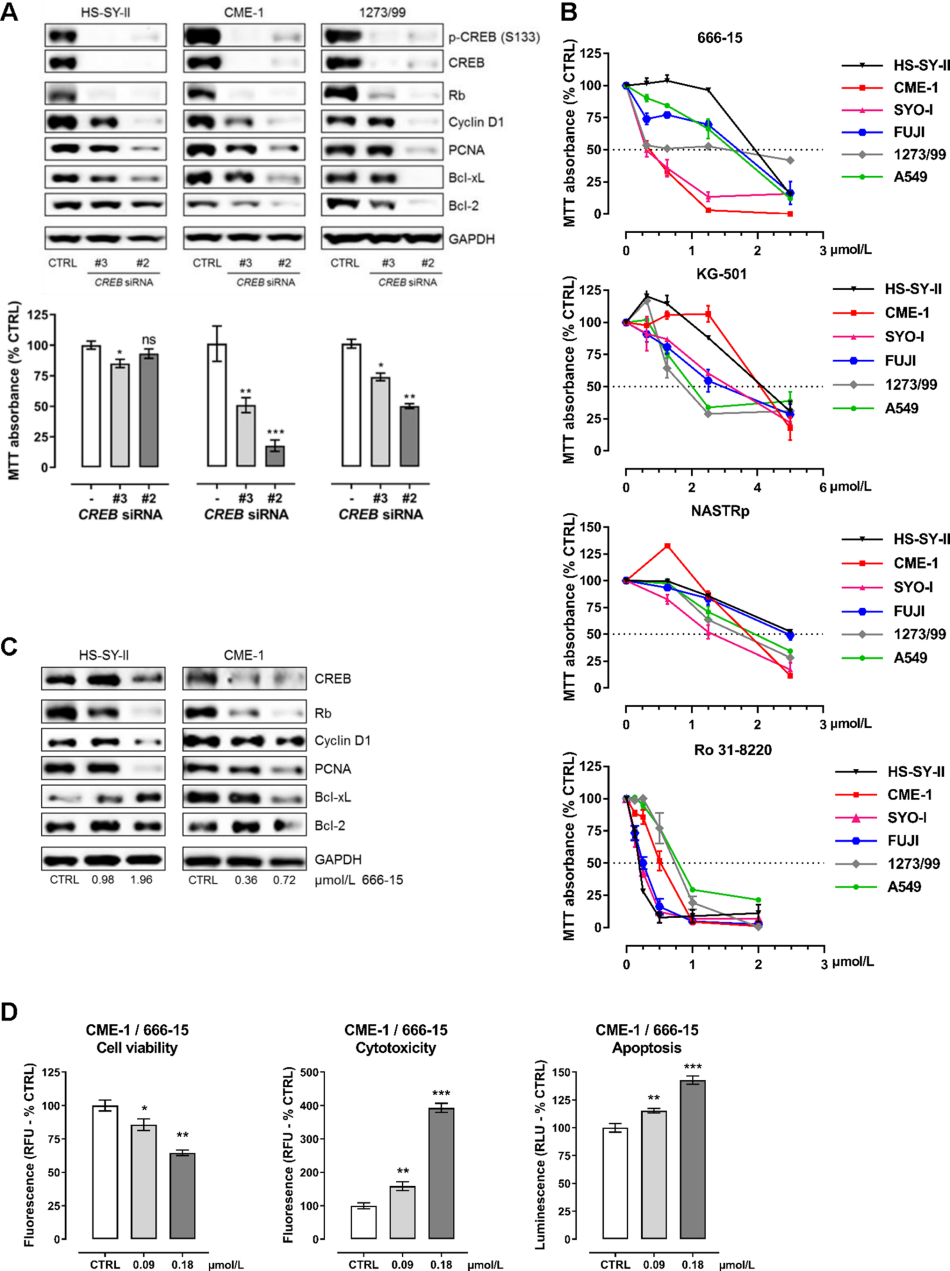
CREB inhibition reduces cell viability and CREB downstream target gene expression in vitro. **A** RNAi-mediated CREB knockdown in three different SySa cell lines is accompanied by reduced downstream target gene expression and reduced cell viability in three SySa cell lines (MTT performed in quintuplicate; results are shown as mean ± SEM). **B** Treatment of five SySa cell lines with increasing concentrations of four different small molecule inhibitors (666-15, KG-501, NASTRp and Ro-318220) significantly reduces cell viability in MTT assays (72 h). A549 cells are included as a positive control. At least three independent experiments were performed, each in quintuplicate; results are shown as mean ± SEM. **C** Treatment of two SySa cell lines with increasing concentrations of 666-15 (0.36-1.96 µmol/L) for 20 h lead to dose-dependent reduction of CREB downstream target gene expression. **D** 666-15 treatment of CME-1 cells results in reduced cell viability and an elevated apoptotic rate (experiment performed in quintuplicate; results shown as mean ± SEM)

To document the functional role of CREB in a non-pharmacological approach, three different SySa cell lines (HS-SY-II, CME-1 and 1273/99) were transfected with siRNAs targeting human *CREB.* RNAi-mediated *CREB* depletion resulted in a reduction of Rb, Cyclin D1, PCNA, Bcl-xL and Bcl-2 protein and mRNA levels (Fig. 3A upper panel, Supplementary Fig. S5), associated with a significant suppression of SySa cell viability (Fig. 3A lower panel).

In order to investigate the biological effects of CREB inhibition, SySa cells were incubated with increasing concentrations (0.13-5 µmol/L) of the CREB inhibitors 666-15, KG-501 and NASTRp and the PKC-inhibitor Ro 31-8220 [43, 46, 50]. In MTT assays, all inhibitors significantly suppressed SySa cell viability in a dose-dependent manner with IC_50_ values ranging from 0.24-4.4 µmol/L (summarized in Table 2). SySa cells were more sensitive to 666-15 (IC_50_:0.36-1.72 µmol/L) treatment compared to NASTRp (IC_50_: 1.17-2.35 µmol/L) or KG501 (IC_50_:1.34-4.40 µmol/L). A549 cells sensitive to CREB inhibition were used as a control [46, 50] and showed similar IC_50_ value ranges compared to all five SySa cell lines (Fig. 3B). Given the higher sensitivity of SySa cells to 666-15 treatment, this substance was selected for subsequent experimentation. Treatment of HS-SY-II and CME-1 cells with 666-15 for 20 h led to reduced Rb, Cyclin D1, PCNA, Bcl-xL and Bcl-2 downstream target protein levels accompanied by decreased CREB protein levels, the latter phenomenon not having been described yet (Fig. 3C). Of note, in HS-SY-II, Bcl-xL was found to be upregulated upon 666-15 treatment, pointing to a complex regulatory network for individual CREB targets. Treatment with KG-501 showed comparable effects on downstream target and CREB protein levels (Supplementary Fig. S4B).

**Table 2:** IC_50_ values

*Compound*	IC_50_ (µmol/L)					
	HS-SY-II	CME-1	SYO-I	FUJI	1273/99	A549
666-15	1.96 ± 0.02	0.36 ± 0.07	0.33 ± 0.22	1.72 ± 0.16	0.71 ± 0.16	1.59 ± 0.12
KG-501	1.79 ± 0.44	1.85 ± 0.29	1.34 ± 0.65	4.40 ± 2.0	1.69 ± 0.31	2.05 ± 0.05
NASTRp	2.35 ± 1.43	1.89 ± 0.06	1.38 ± 0.64	2.81 ± 0.75	1.17 ± 0.73	1.91 ± 0.11
Ro 31-8220	0.24 ± 0.07	0.78 ± 0.38	0.23 ± 0.10	0.25 ± 0.04	0.90 ± 0.0	0.76 ± 0.01

Cytotoxic effects on viability of SySa cell lines (HS-SY-II, CME-1, SYO-I, FUJI, 1273/99) and the CREB-sensitive lung adenocarcinoma cell line A549 were assessed by MTT assays. Results are presented as mean ± SEM of at least three independent experiments performed in quintuplicates

To assess the anti-proliferative effects of 666-15 treatment in SySa cells, ApoTox-Glo Triplex assays were performed to uncover changes in SySa cell viability, cytotoxicity and apoptosis. Incubation of CME-1, HS-SY-II and SYO-I cells with 666-15 for 20 h resulted in significant suppression of cell viability accompanied by a significant induction of apoptosis (Fig. 3D, Supplementary Fig. S6).

### In vivo efficacy of 666-15 in SySa cell line-based chorioallantoic membrane (CAM) assays and mouse xenografts

**Fig. 4:**
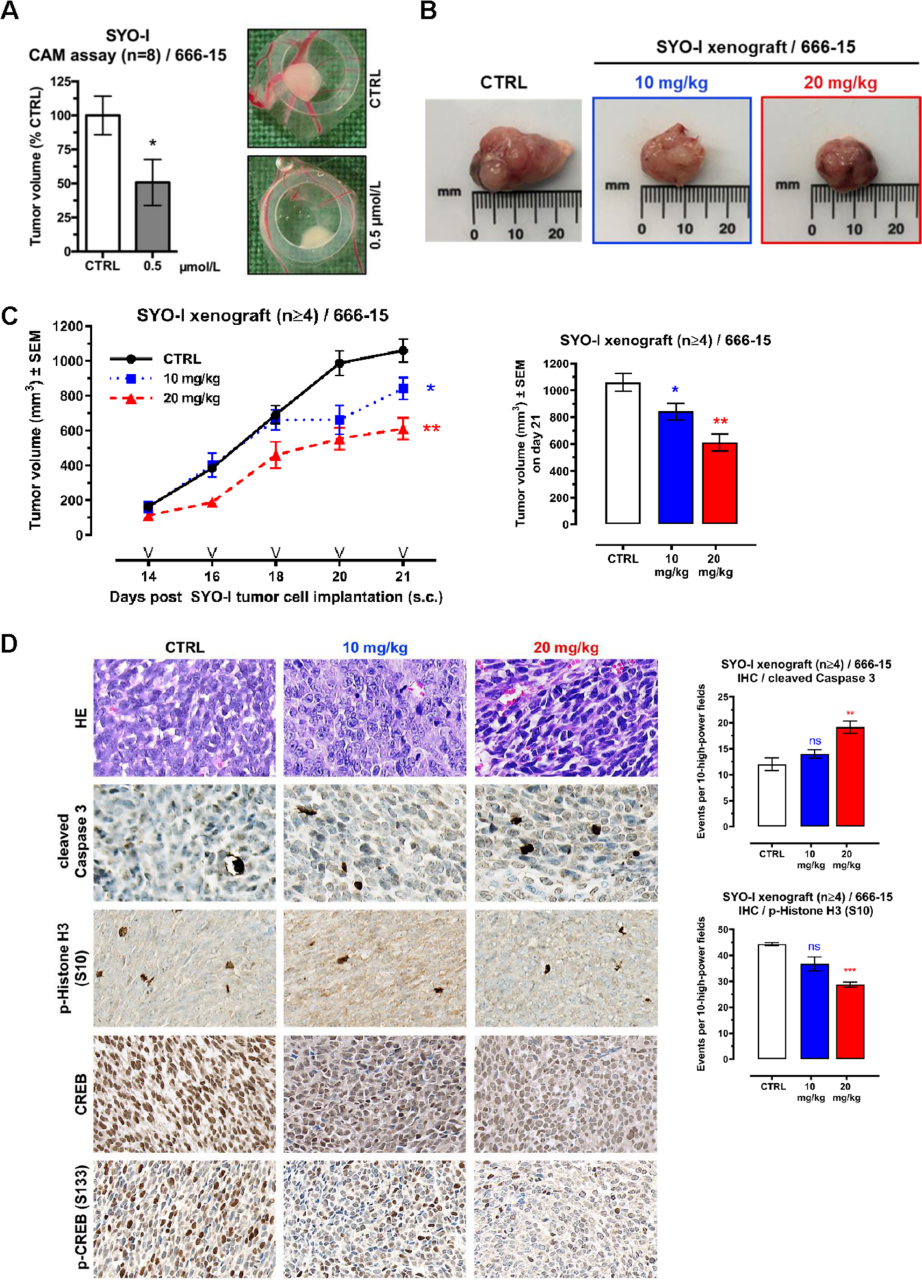
In vivo efficacy of 666-15 in SySa cell line-based chorioallantoic membrane and mouse xenografts. **A** SySa tumor growth on chicken CAM following treatment with 0.5 µmol/L 666-15; at least 8 tumors were included in the analysis (left). Representative examples are shown (right). **B** Representative photographs of murine SYO-I xenografts treated with different doses of 666-15. **C** Left: SYO-I xenograft tumor volumes (mm^3^) measured every other day shown as mean ± SEM for every treatment arm for the period of treatment with 666-15. “V” indicates the days of treatment. Right: mean tumor volume (± SEM) for each treatment arm (Vehicle CTRL, 10 mg/kg 666-15 or 20 mg/kg 666-15) on day 21. A significantly reduced tumor volume (mm^3^) is observed in mice treated with 666-15 (10 or 20 mg/kg/d). **D** Left: representative IHC staining (HE, cleaved Caspase 3, p-Histone H3 (S10), total CREB, and p-CREB (S133)) for each treatment arm (CTRL, 10 mg/kg 666-15 or 20 mg/kg 666-15). Right: 666-15 treatment leads to an increase of cleaved Caspase 3 positive cells along with a decrease of p-Histone H3 (S10) positive cells as evaluated in 10 high power fields

As a final step of our preclinical evaluation of the efficacy of CREB inhibition on SySa tumor growth, we applied two different in vivo models. First, we performed chick CAM assays xenografting SYO-I cells to initiate SySa tumor formation. We found that topical administration of 666-15 (0.5 µmol/L) led to a significant inhibition of SYO-I tumor growth and a reduction in tumor volume compared to the vehicle-treated control group (Fig. 4A). Second, SYO-I cells were subcutaneously xenografted into the lower flanks of NSG mice that were either treated with 666-15 or vehicle CTRL. Intraperitoneal administration of 666-15 (every other day) resulted in a significant and dose-dependent suppression of SYO-I tumor growth and a reduction in tumor volume compared to the DMSO vehicle control group (Fig. 4B, C). No negative side effects of treatment with 666-15 on body weights were observed (Supplementary Fig. S7). Immunohistochemical analyses of p-Histone H3 (S10, marker of mitotic activity) and cleaved Caspase 3 (marker of apoptosis) showed (i) a significant reduction of mitotic activity accompanied by (ii) induction of apoptosis and (iii) reduced total CREB protein and (iv) p-CREB (S133) phosphorylation levels in SySa xenografts treated with 666-15 compared to the vehicle-treated control group (Fig. 4D).

## Discussion

Synovial Sarcoma (SySa) is genetically characterized by a specific reciprocal translocation t(X;18)(p11;q11), which has convincingly been defined as the major oncogenic driver of the disease, i.e., the SS18-SSX transcriptional dysregulator [1, 2, 51]. SS18-SSX is known to interact with chromatin remodelling complexes, in line with the “original” functions of SS18 and SSX [15, 16]. However, the exact molecular realization of the oncogenic stimulus of SS18-SSX in terms of oncogenic signals is still incompletely understood, though aberrant patterns of the IGF-IR/PI3K/Akt, SRC, WNT and Hippo pathways have previously been described [11–14, 39]. The prognosis of SySa is poor in case of metastasized disease, and current treatment options are limited to surgery, conventional chemotherapy and radiotherapy. So, innovative therapeutic approaches are required. Given the notorious difficulty to target the fusion protein itself, functional insights into SS18-SSX-shaped tumor biology are essential to decipher druggable tumor vulnerabilities. The transcription factor CREB plays an essential role in development and cell physiology, impacting the expression of about 4000 genes. In diverse types of cancer, including acute myeloid leukaemia, prostate cancer, breast cancer and non-small lung cancer [33–36], (aberrantly) activated CREB has been associated with increased cellular proliferation. Although little is known about the functional role of CREB in sarcomas, pathogenic translocations between *EWSR1* and *CREB* or its homologue *ATF1* in clear cell sarcoma, another fusion-driven mesenchymal neoplasia, point to a major functional role in mesenchymal tumorigenesis [37, 38]. Based on results of Phospho-Kinase arrays performed in SySa cells we analysed the functional role of CREB in SySa.

In a large cohort of tumor specimens and a representative set of SySa cell lines, we detected a high prevalence of p-CREB (S133) expression, associated with concordant expression of its downstream targets Rb, Cyclin D1, PCNA, Bcl-xL and Bcl-2 in a large subset of primary SySa and all tested SySa cell lines. By assessing the impact of SS18-SSX on CREB signals in two model systems, we found CREB S133 phosphorylation and expression to be functionally dependent on the chimeric SS18-SSX fusion protein, both in RNAi-based experiments in SySa cells and in SS18-SSX overexpression experiments in SCP-1 mesenchymal stem cells. As activation of the IGF-IR-signaling axis is a well-known feature of SySa [11], we wondered whether IGF-II signals may contribute to aberrant CREB activation. We found that phosphorylation of CREB (S133) in SySa cells was induced by IGF-II and reduced through RNAi-mediated *IGF-IR* knockdown or the IGF-IR inhibitor BMS-754807. Our findings, therefore, suggest that in SySa, IGF-IR/PI3K/Akt signals may be involved in CREB regulation, as has previously also been described in other malignancies [26, 52]. Still, IGF-IR-dependent signals are certainly not unique in activating CREB in SySa, given the known role of other kinases such as PKA, PKC, CaMKII, CaMKIV or p90RSK [26–30] in S133 phosphorylation of CREB. Targeting CREB, either by RNAi or through pharmacological inhibition, led to a significant and dose-dependent effect on SySa cell growth in MTT assays, coupled with the expected regulation of CREB downstream targets which are known to be involved in many different processes, including apoptosis (Bcl-xL and Bcl-2), cellular proliferation (PCNA) and cell cycle progression (Rb and Cyclin D1). Though pharmacological inhibition of kinases acting upstream of CREB, e.g. with Ro 31-8220, as reported previously [47, 50], efficiently inhibits CREB (S133) phosphorylation, this approach lacks specificity. As inhibition of the interaction between CREB and the CRE-element is technically challenging with suitable substances not being available [53], we set out to test interaction between the KID-domain of CREB and the KIX-domain of CBP, which is elementary for the induction of downstream target gene expression. Significant and concordant biological activity of all substances tested was detected, and we could show that, mechanistically, the most potent inhibitor 666-15 led to an induction of apoptosis in CME-1, HS-SY-II and SYO-I cells. These data in SySa fit well with observations from other tumors as summarized by Xiao and colleagues [54]. Importantly, it has been shown that normal cells are not sensitive to CREB inhibition [55, 56]. Consistent with our in vitro results, topical administration of 666-15 to SYO-I CAM xenografts led to a significant suppression of SySa tumor growth in vivo. This finding could be translated to SYO-I NSG mouse xenografts, in which we observed significant therapeutic effects of treatment with 666-15, without severe side effects being observed (e.g. reduction in body weight). In the tumor explants, IHC staining revealed (i) an induction of cleaved Caspase 3-positive cells due to 666-15 treatment, associated with (ii) reduced phosphorylation of Histone H3 S10, (iii) reduced total CREB protein and (iv) p-CREB (S133) phosphorylation levels.

Based on our preclinical findings, indicating a major role of CREB in SySa tumorigenesis, these rare mesenchymal tumors appear to functionally join clear cell sarcoma, in which *EWSR1*-*CREB* or *EWSR1*-*ATF1* gene fusions drive sarcomagenesis, directly involving CREB within the fusion protein [37, 38]. Though, obviously there is a considerable diversity of biological effects being associated with CREB activation in different soft tissue tumors, CREB dysregulation may be regarded as a common theme in a subset of mesenchymal tumors enabling efficient “cross-entity” molecular targeting strategies.

In conclusion, our preclinical study indicates that CREB phosphorylation and activity represent a common pattern in SySa tumorigenesis, being functionally dependent on the chimeric SS18-SSX fusion protein. Disruption of CREB-mediated signals via  small molecule inhibitors may provide an effective and novel therapeutic approach for the treatment of SySa that warrants further investigation.

## Supplementary Information

Below is the link to the electronic supplementary material.

Supplementary file1 (PDF 961 kb)

Supplementary file2 (PDF 3919 kb)

## Data Availability

All data are included in the original figures and tables.
