# Supplementary material for: SS18-SSX drives CREB activation in synovial sarcoma
Source: Cell Oncol (Dordr). 2022 May 12;45(3):399–413. doi: 10.1007/s13402-022-00673-w (PMC9187574; doi:10.1007/s13402-022-00673-w)
Supplement: Supplementary file 1 — Supplementary file1 (PDF 961 kb) [file 13402_2022_673_MOESM1_ESM.pdf]

SS18-SSX drives CREB activation in Synovial Sarcoma

Magdalene Cyra[1,2*], Miriam Schulte[1,2*], Ruth Berthold[1,2], Lorena Heinst[1,2], Esther-Pia Jansen[1,2], Inga Grünewald[1,2], Sandra Elges[2], Olle Larsson[3], Christoph Schliemann[4], Konrad Steinestel[2,5], Susanne Hafner[6], Thomas Simmet[6], Eva Wardelmann[2], Sareetha Kailayangiri[7], Claudia Rossig[7], Ilka Isfort[1,2,#], Marcel Trautmann[1,2,#], and Wolfgang Hartmann[1,2,#]

[1] Division of Translational Pathology, Gerhard-Domagk-Institute of Pathology, Münster University Hospital, Münster, Germany

[2] Gerhard-Domagk-Institute of Pathology, Münster University Hospital, Münster, Germany

[3] Departments of Oncology and Pathology, The Karolinska Institute, Stockholm, Sweden

[4] Department of Medicine A, Hematology, Oncology and Respiratory Medicine, Münster University Hospital, Münster, Germany

[5] Institute of Pathology and Molecular Pathology, Bundeswehrkrankenhaus Ulm, Ulm, Germany

[6] Institute of Pharmacology of Natural Products and Clinical Pharmacology, Ulm University, Ulm, Germany

[7] Department of Pediatric Hematology and Oncology, University Children´s Hospital Münster, Münster, Germany

[*] share first authorship, [#] share senior authorship

Correspondence

Wolfgang Hartmann and Marcel Trautmann, Division of Translational Pathology, Gerhard-Domagk-Institute of Pathology, Münster University Hospital Münster, Albert-Schweitzer-Campus 1, D17, 48149 Münster, Germany. Phone: +49-251-83-58479 and -57623; Fax: +49 251-83-57559. E-mail: wolfgang.hartmann@ukmuenster.de and marcel.trautmann@ukmuenster.de

Running title

CREB activity in synovial sarcoma

Keywords

Synovial sarcoma, SS18-SSX, CREB, 666-15, BMS-754807

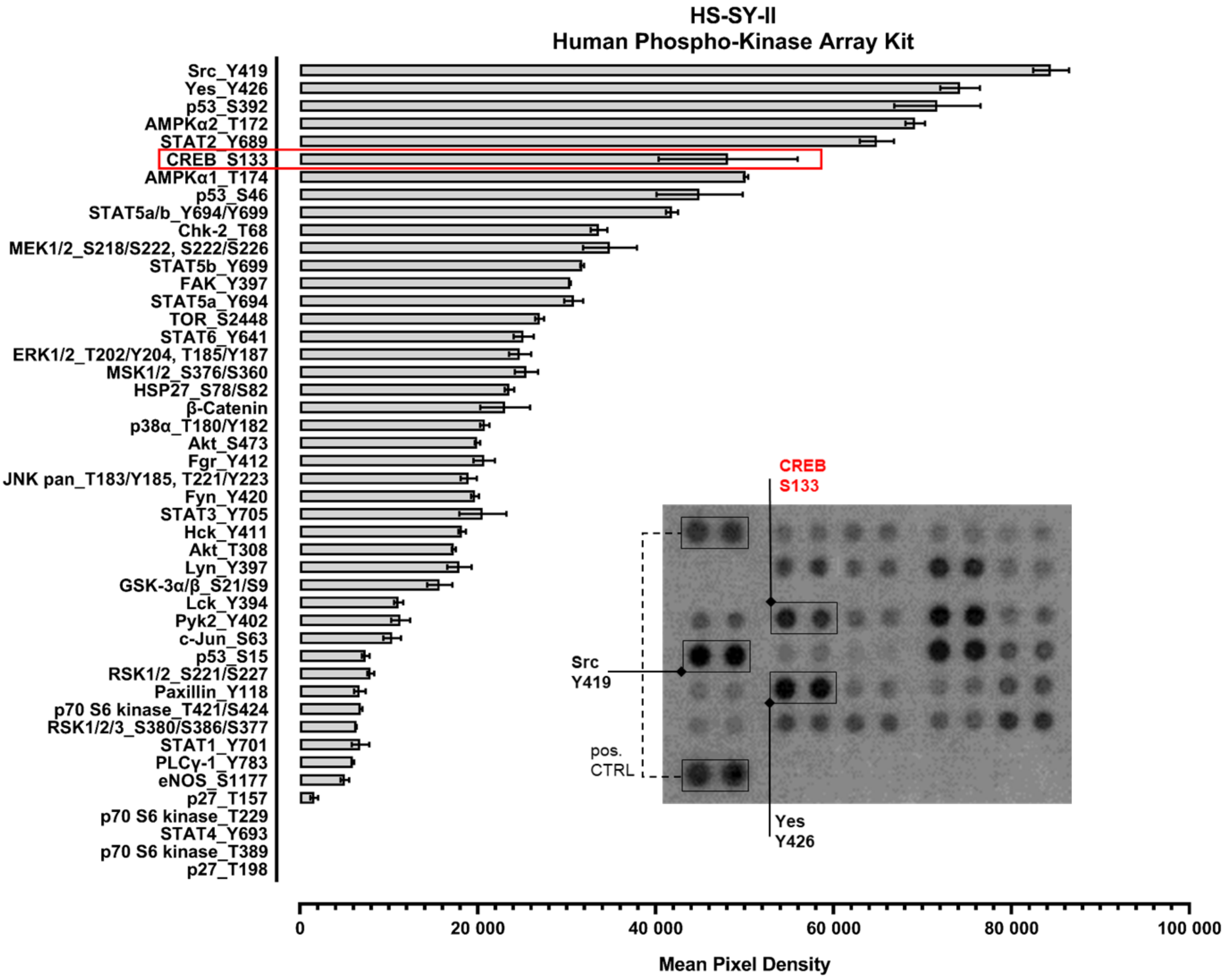

Supplementary Figure S1.

The profile of 46 phospho-kinases indicates activating CREB S133 phosphorylation to be prominent in HS-SY-II synovial sarcoma cells along with the known activation of SRC family kinases.

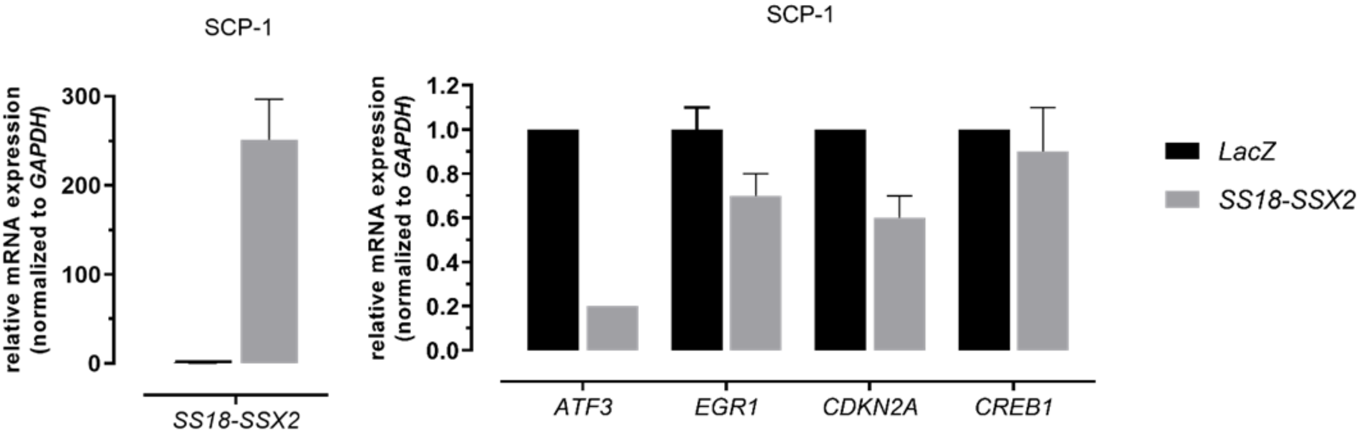

Supplementary Figure S2.

Transient expression of *SS18-SSX* in SCP-1 mesenchymal stem cells regulates *ATF3*, *EGR1*, and *CDKN2A* target gene expression (*LacZ*, control). *CREB* mRNA levels do not increase upon *SS18-SSX* overexpression in SCP-1 cells.

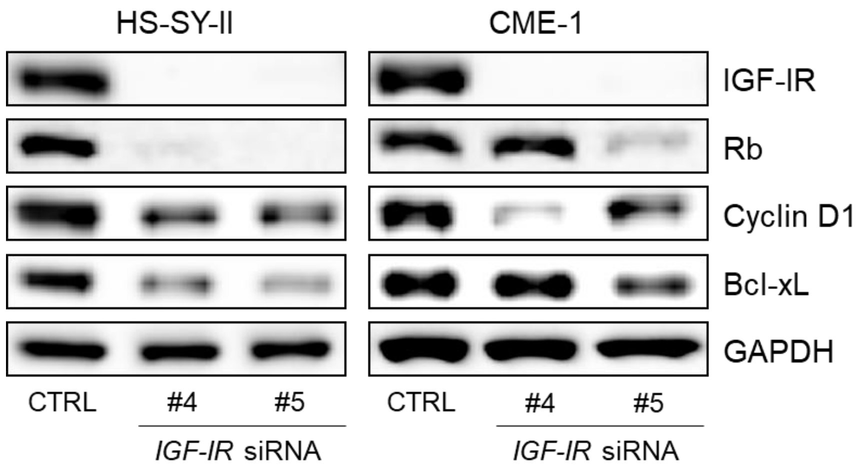

Supplementary Figure S3.

RNAi-mediated *IGF-IR* depletion is associated with reduced protein expression of CREB downstream targets including Rb, Cyclin D1 and Bcl-xL in HS-SY-II and CME-1 cells.

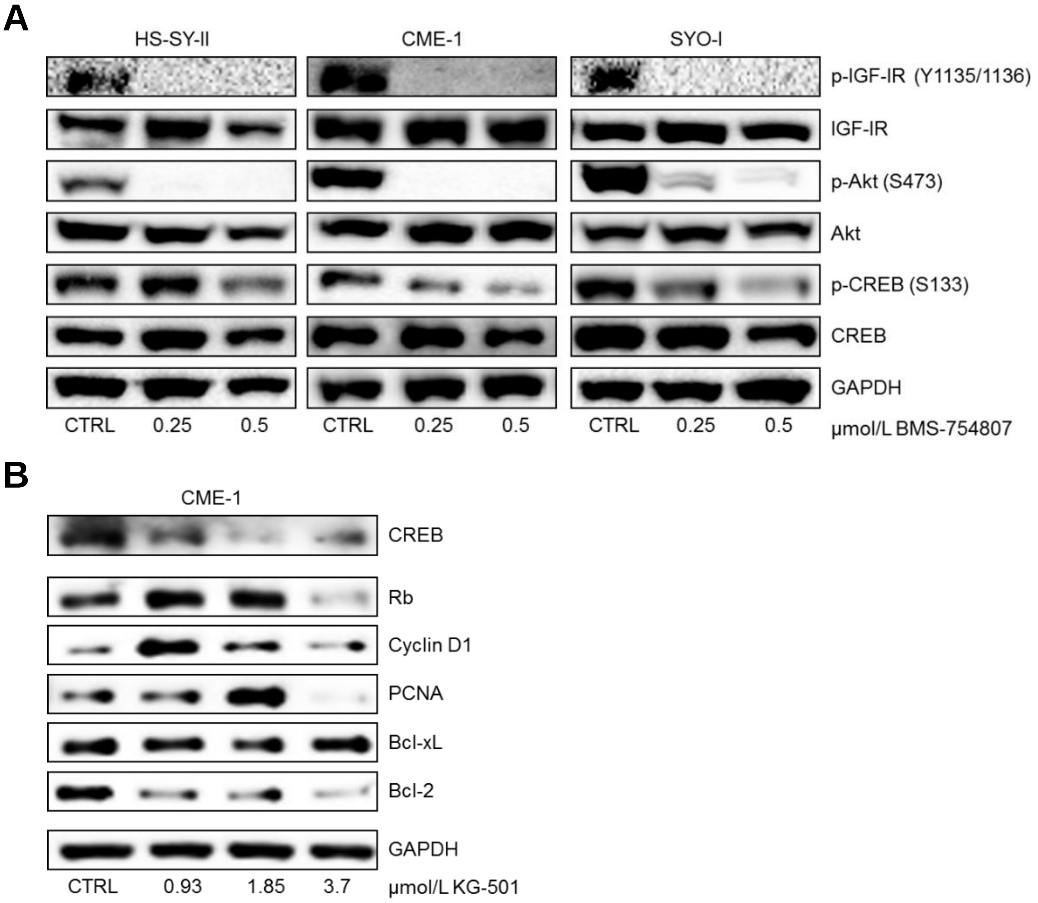

Supplementary Figure S4.

(A) Treatment of SySa cell lines with the IGF-IR kinase inhibitor BMS-754807 (0.25-0.5 µmol/L) leads to dose-dependent reduction of phosphorylation of IGF-IR (Y1135/1136), Akt (S473) and CREB (S133). **(B)** Immunoblot of CME-1 protein lysates after 6 hours of KG-501 treatment showing reduction of protein expression of both, CREB and CREB downstream targets.

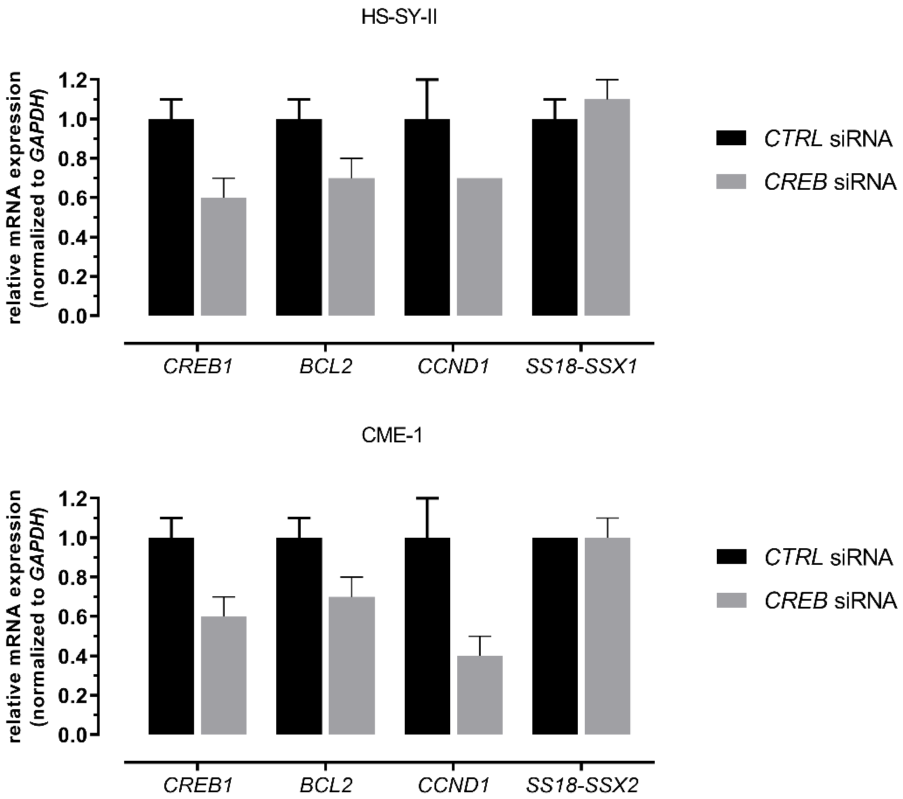

Supplementary Figure S5.

RNAi-mediated *CREB* depletion influences mRNA expression of CREB downstream target gene expression, as indicated by reduced *BCL2* and *CCND1* mRNA levels in HS-SY-II and CME-1 cells.

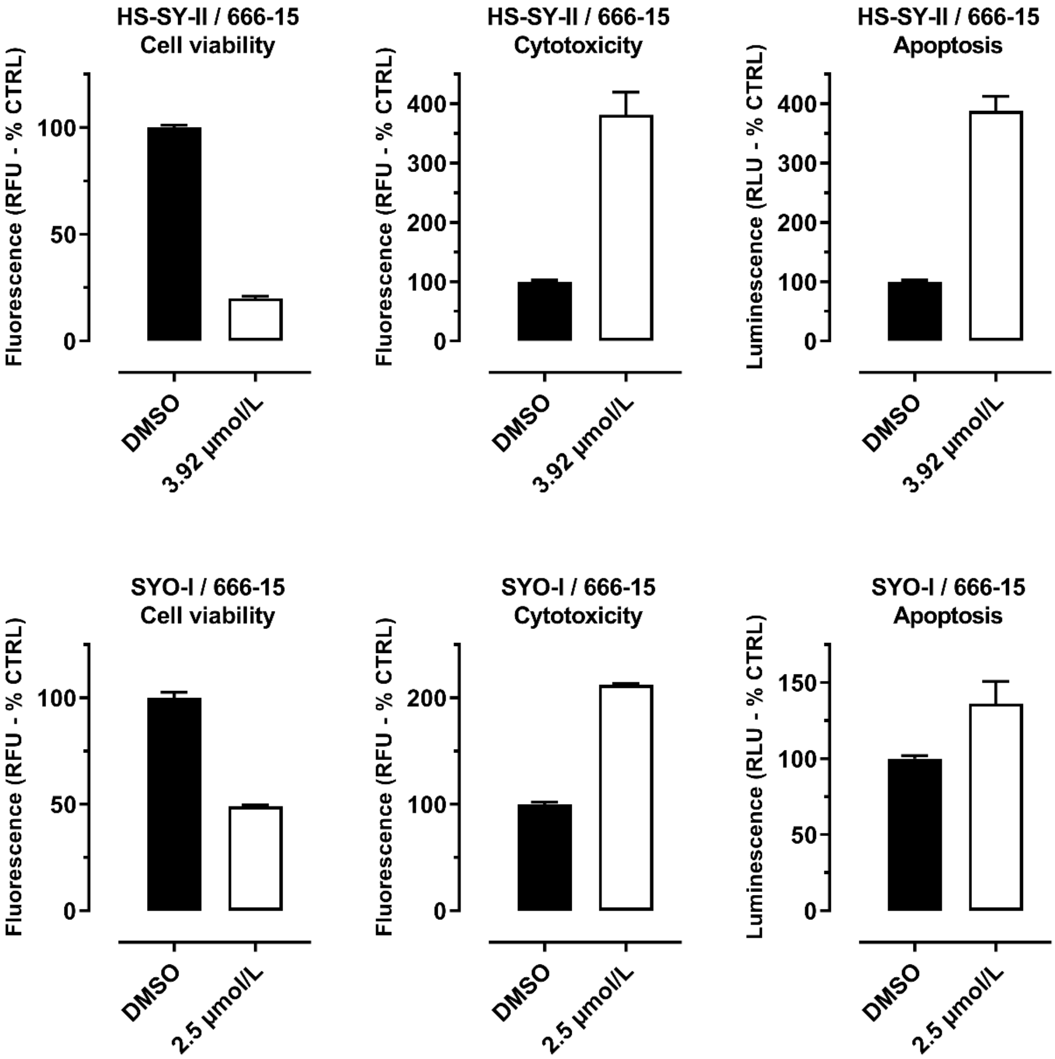

Supplementary Figure S6.

ApoTox-Glo Triplex assays were performed to uncover changes in SySa cell viability, cytotoxicity, and apoptosis. Incubation of HS-SY-II and SYO-I cells with 666-15 resulted in suppression of cell viability accompanied by an induction of apoptosis (experiments performed in quintuplicate; results shown as mean ± SEM).

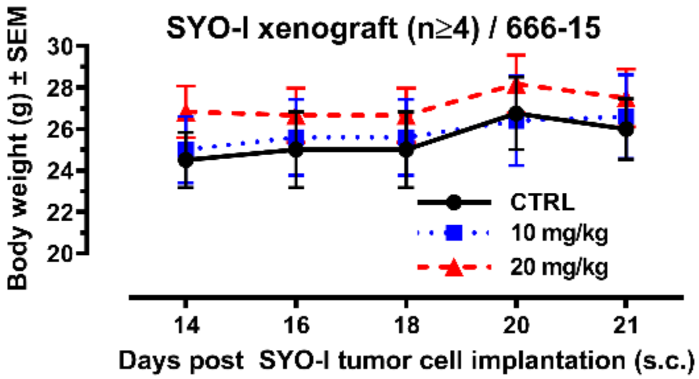

Supplementary Figure S7.

Mean body weight (± SEM) of treated mice shown for each treatment arm (Vehicle CTRL, 10 mg/kg 666-15 or 20 mg/kg 666-15) over 7 days starting with the first day of treatment.
