# Supplementary material for: SS18-SSX drives CREB activation in synovial sarcoma
Source: Cell Oncol (Dordr). 2022 May 12;45(3):399–413. doi: 10.1007/s13402-022-00673-w (PMC9187574; doi:10.1007/s13402-022-00673-w)
Supplement: Supplementary file 2 — Supplementary file2 (PDF 3919 kb) [file 13402_2022_673_MOESM2_ESM.pdf]

Figure 1B

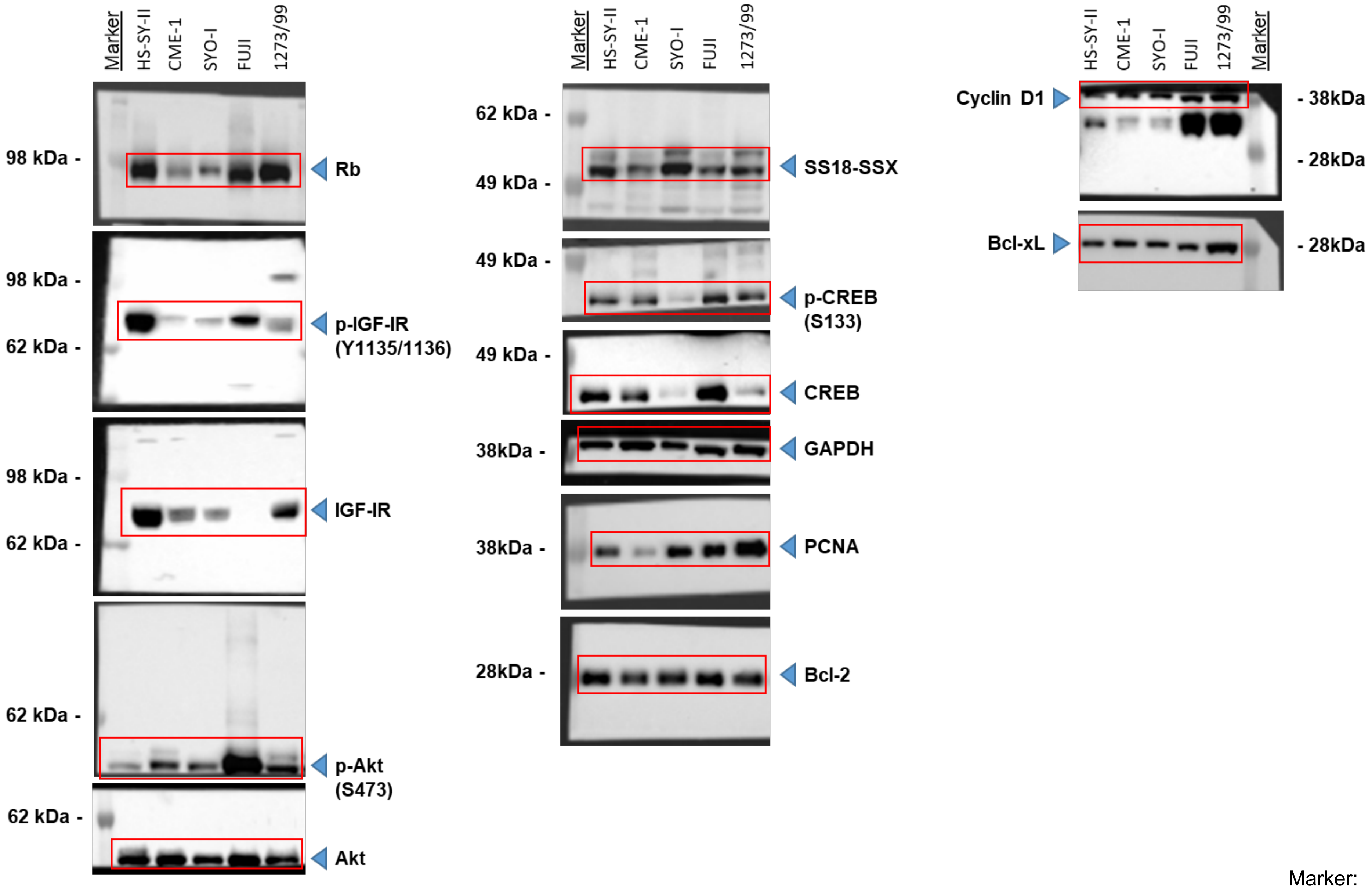

Marker:
SeeBlue Plus2 Pre-Stained Protein Standard (Thermo Fisher Scientific)

Figure 2A

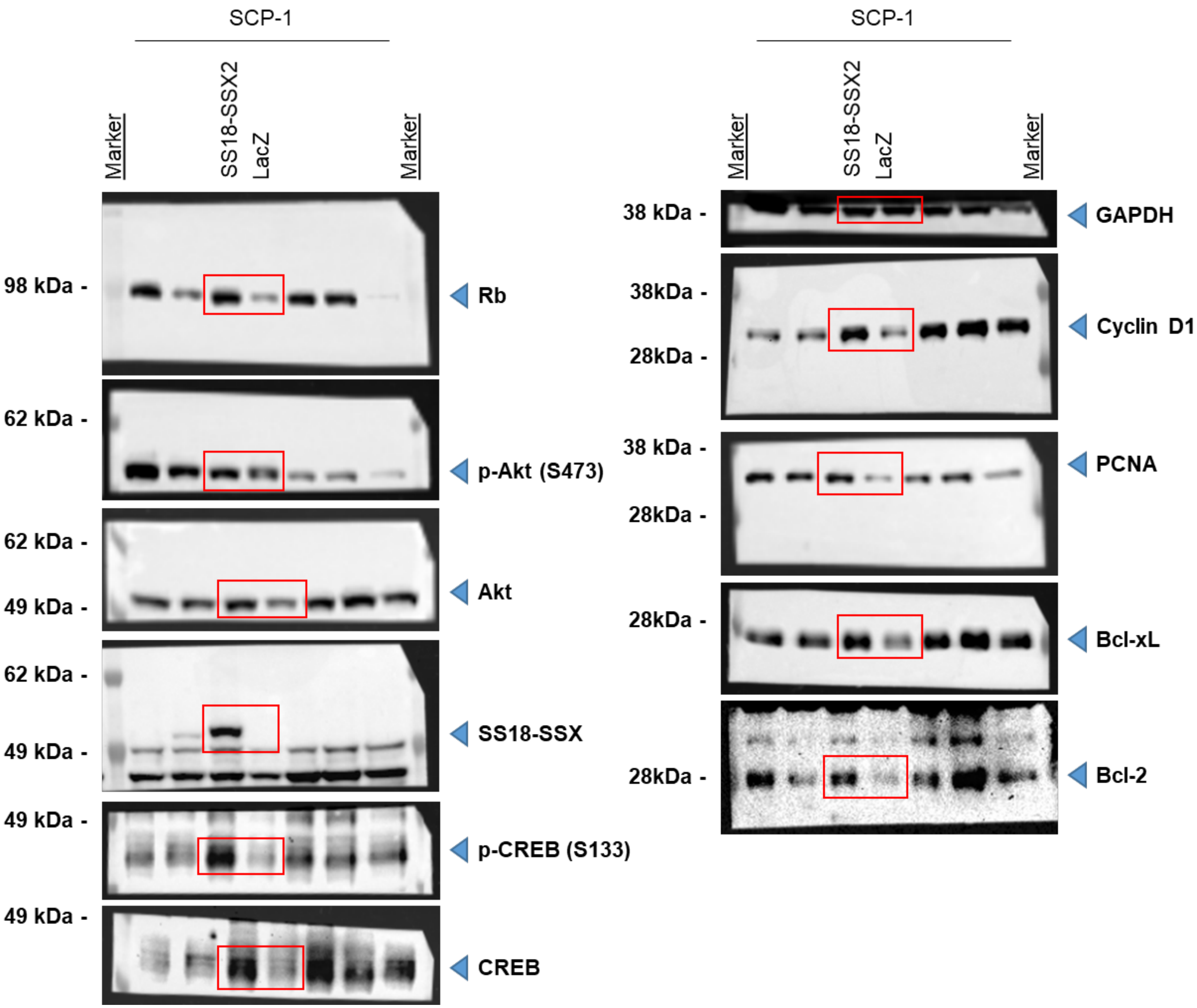

Marker:
SeeBlue Plus2 Pre-Stained Protein Standard
(Thermo Fisher Scientific)

Figure 2B

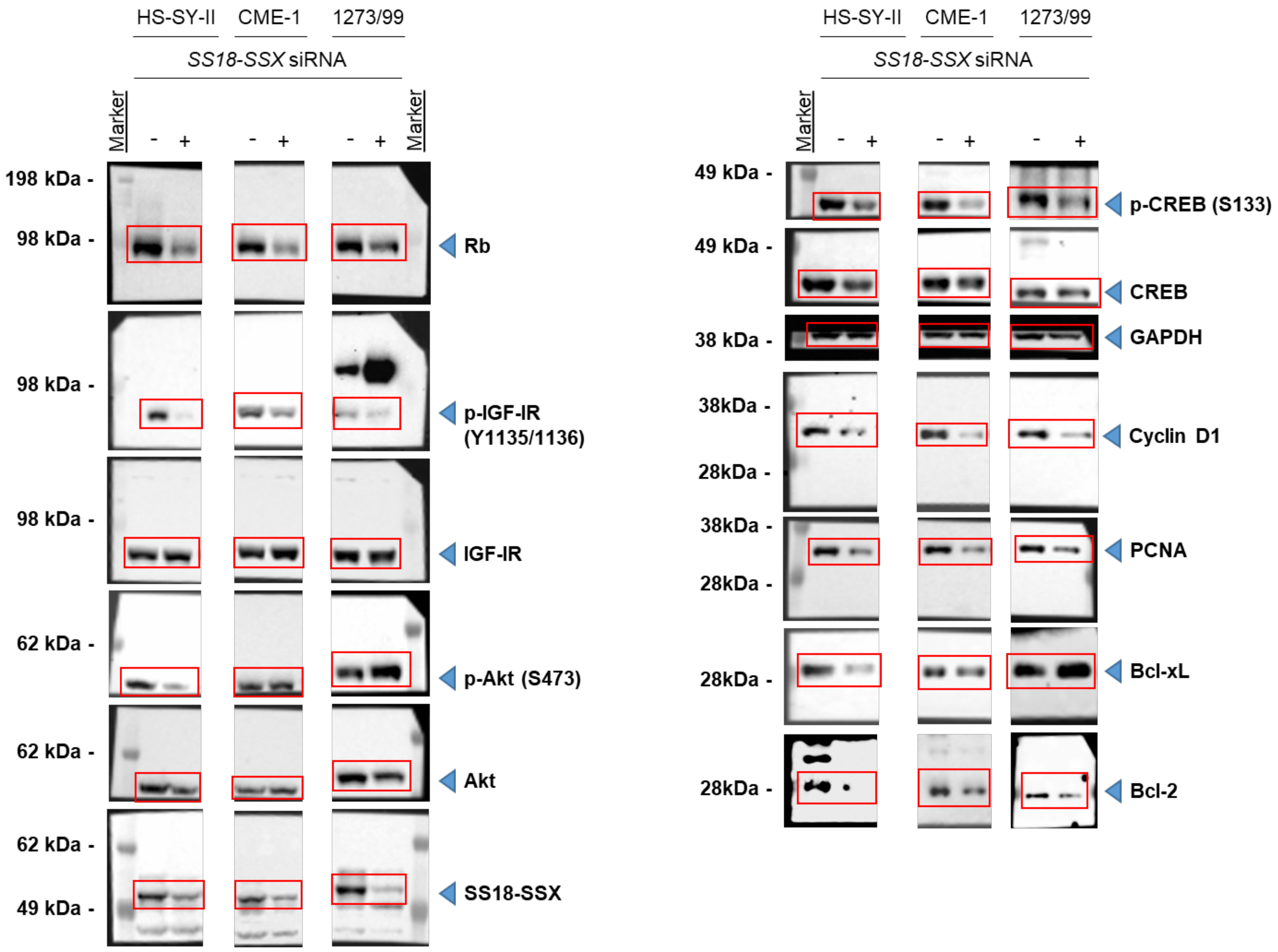

Marker:
SeeBlue Plus2 Pre-Stained Protein Standard
(Thermo Fisher Scientific)

Figure 2C

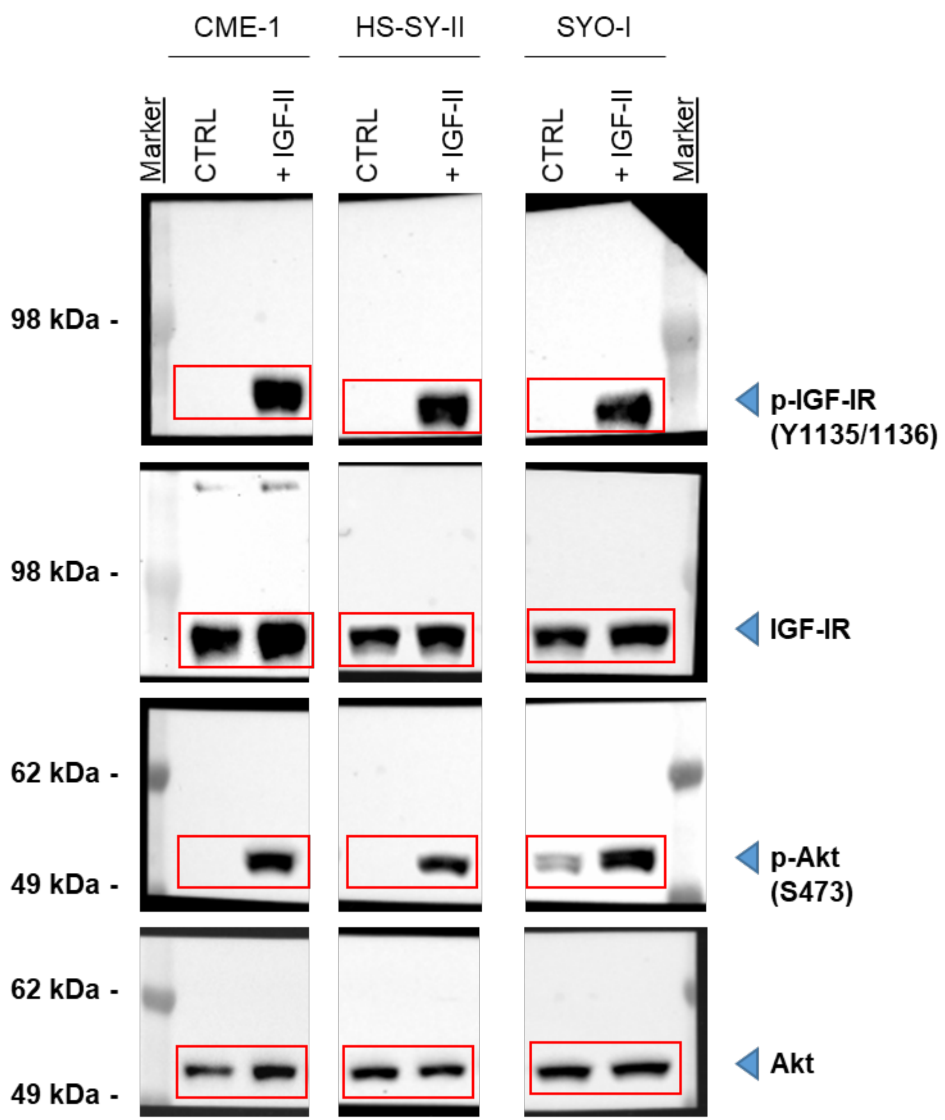

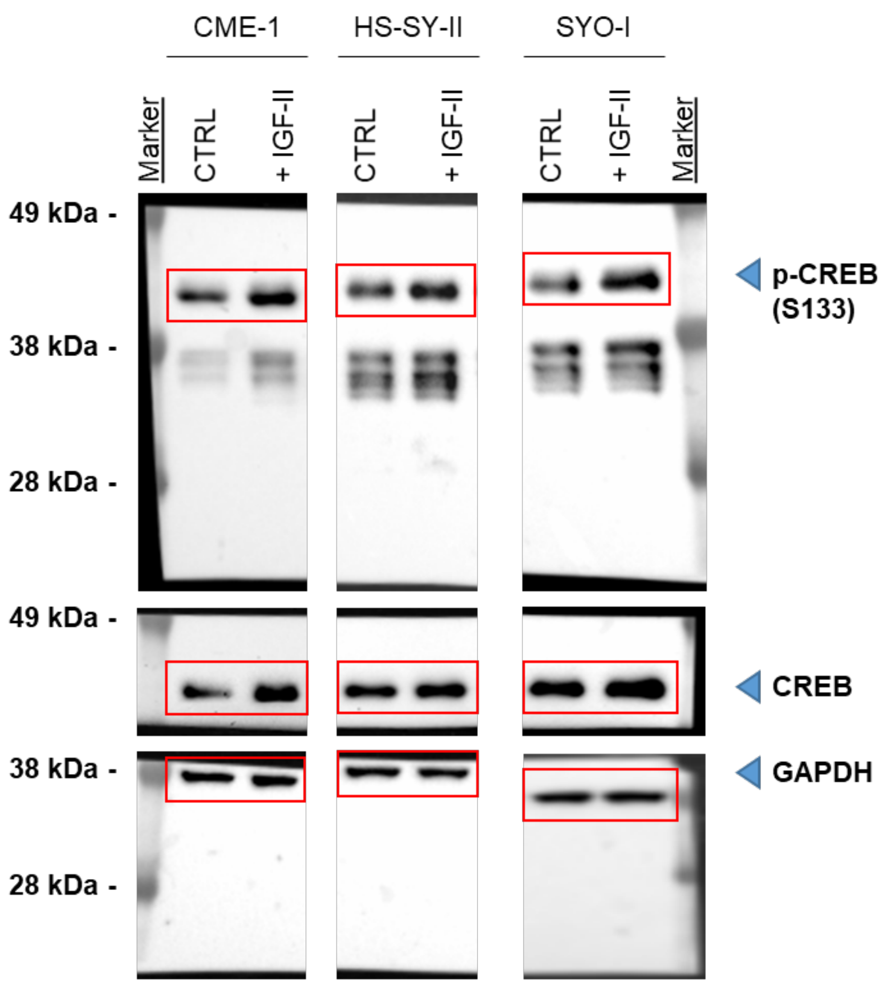

Marker:
SeeBlue Plus2 Pre-Stained Protein Standard
(Thermo Fisher Scientific)

Figure 2D

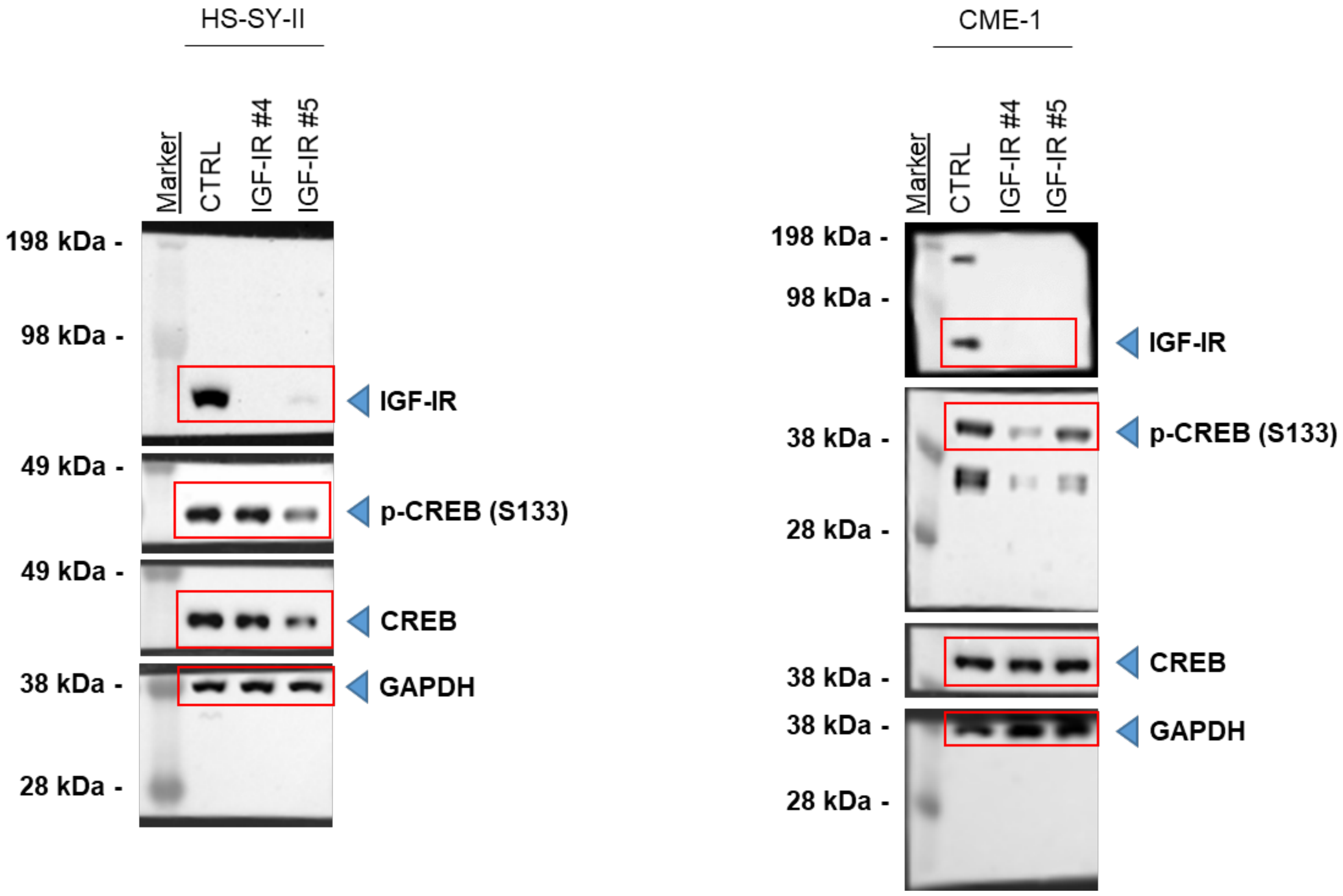

Marker:
SeeBlue Plus2 Pre-Stained Protein Standard
(Thermo Fisher Scientific)

Figure 3A

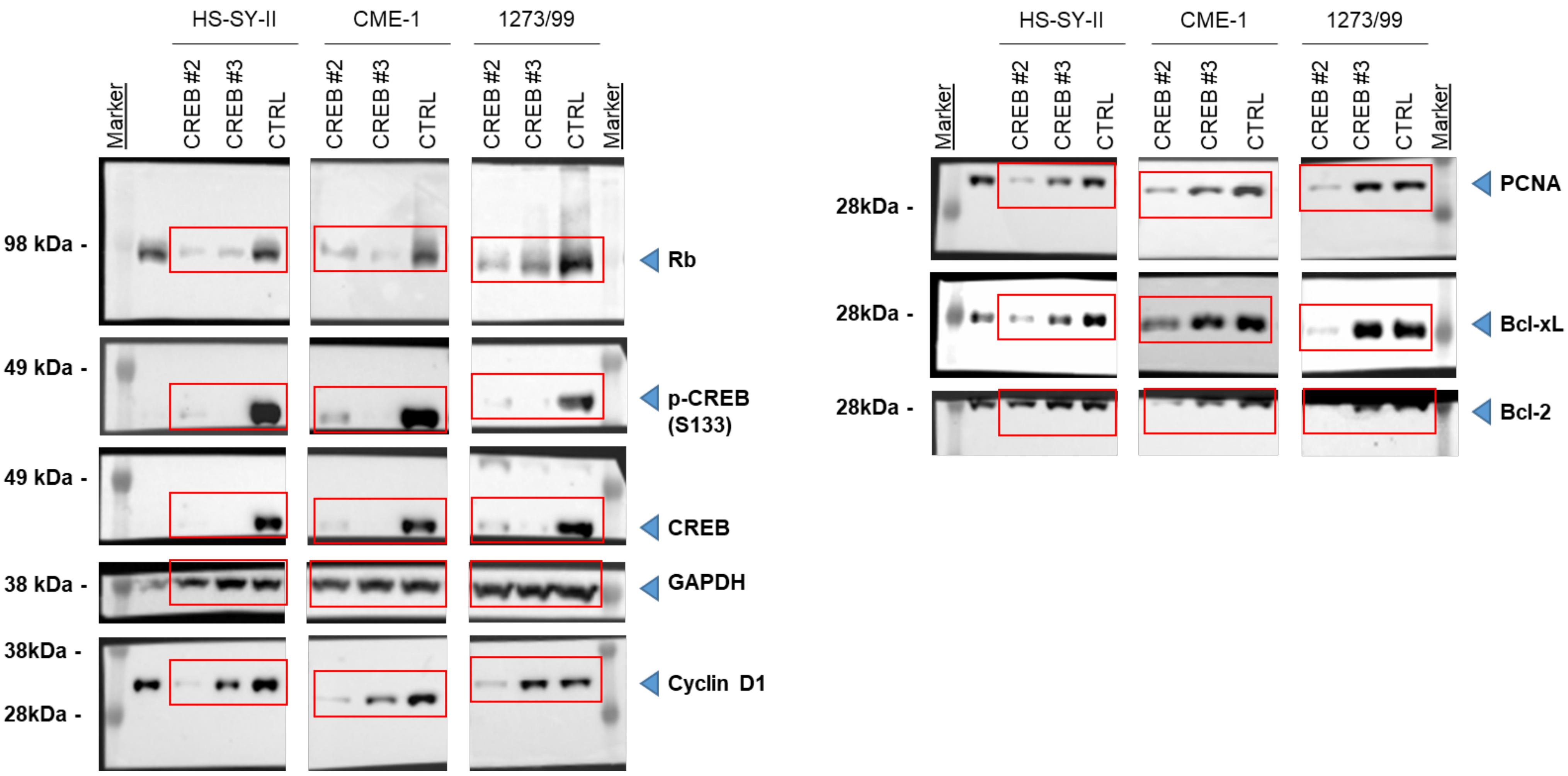

Marker:
SeeBlue Plus2 Pre-Stained Protein Standard
(Thermo Fisher Scientific)

Figure 3C

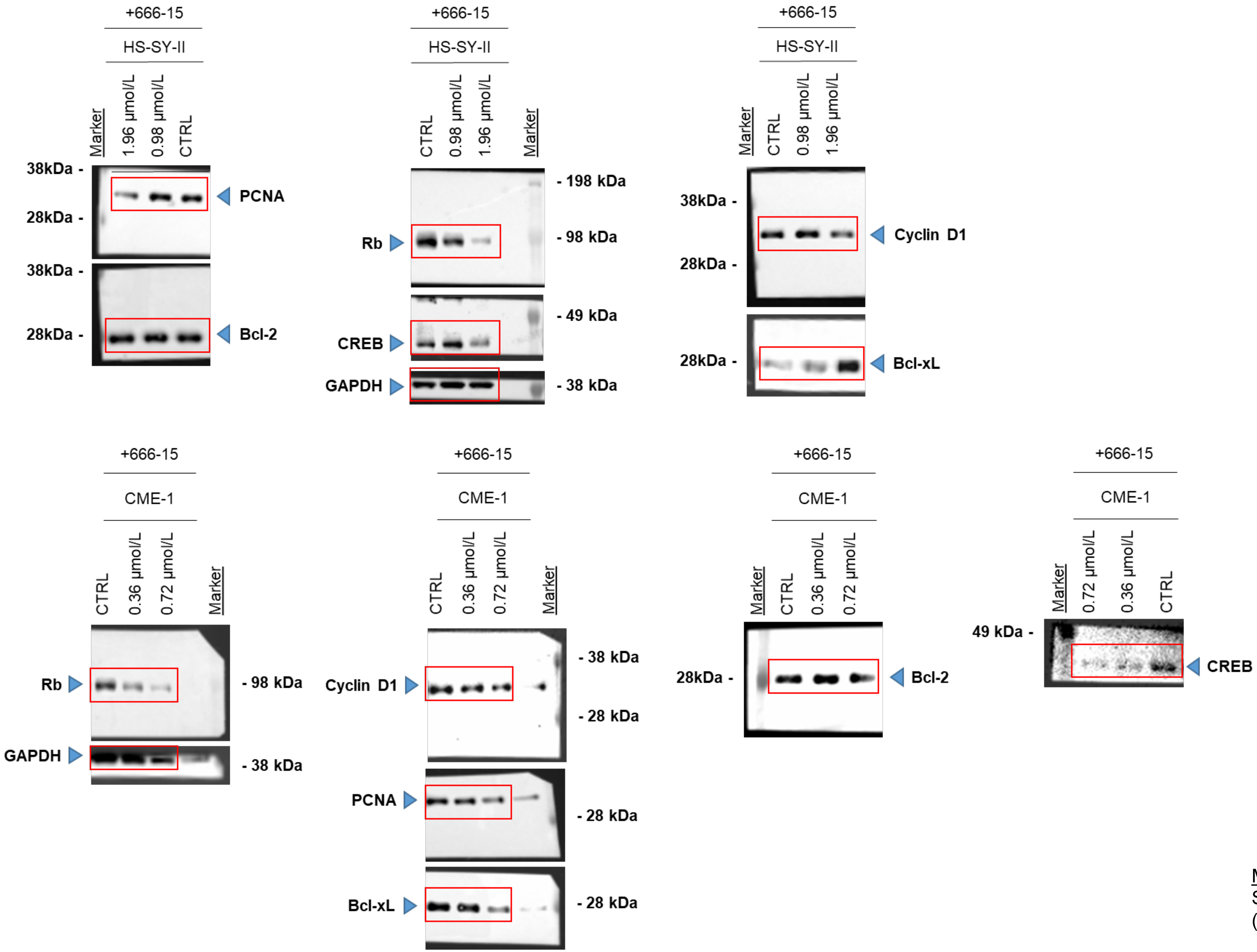

Marker:
SeeBlue Plus2 Pre-Stained Protein Standard
(Thermo Fisher Scientific)

Supplementary Figure S3

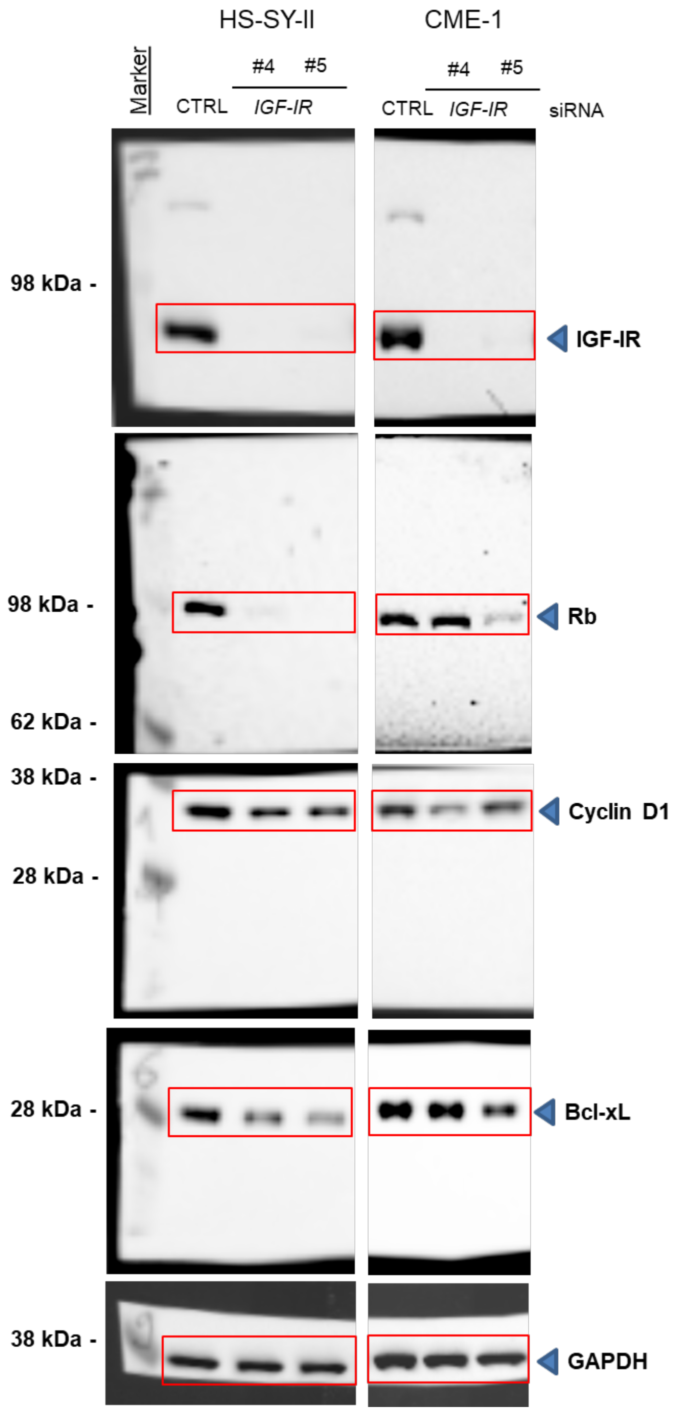

Marker:
SeeBlue Plus2 Pre-Stained Protein Standard
(Thermo Fisher Scientific)

Figure S4 A

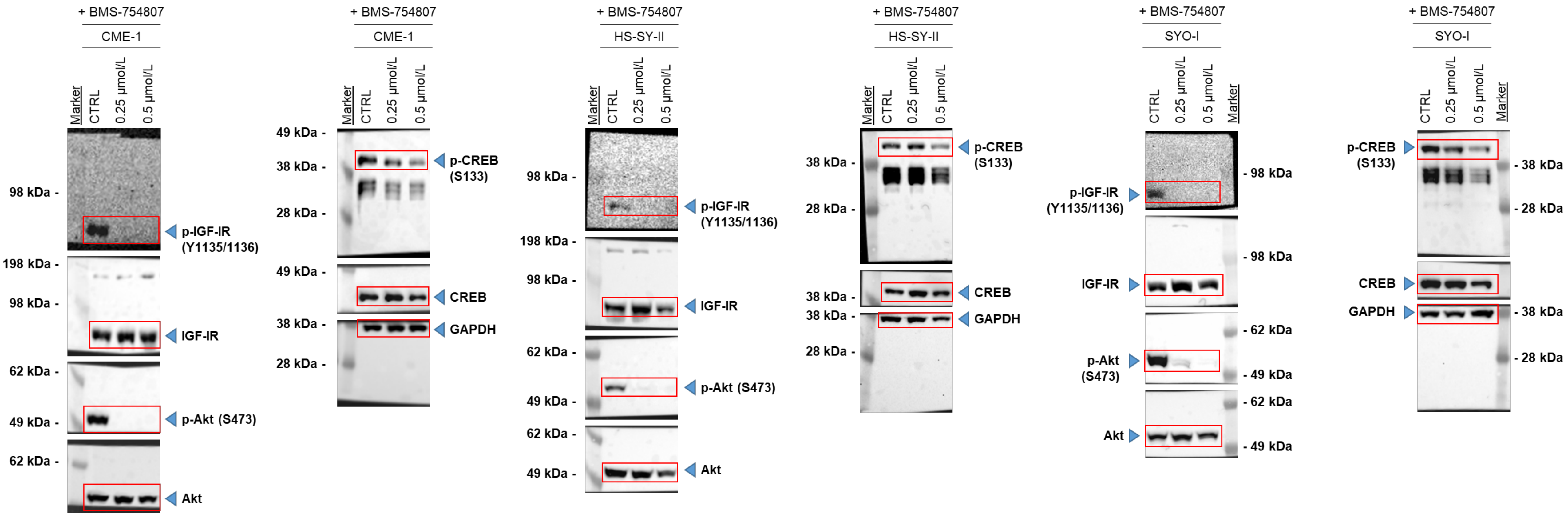

Marker:
SeeBlue Plus2 Pre-Stained Protein Standard
(Thermo Fisher Scientific)

Figure S4 B

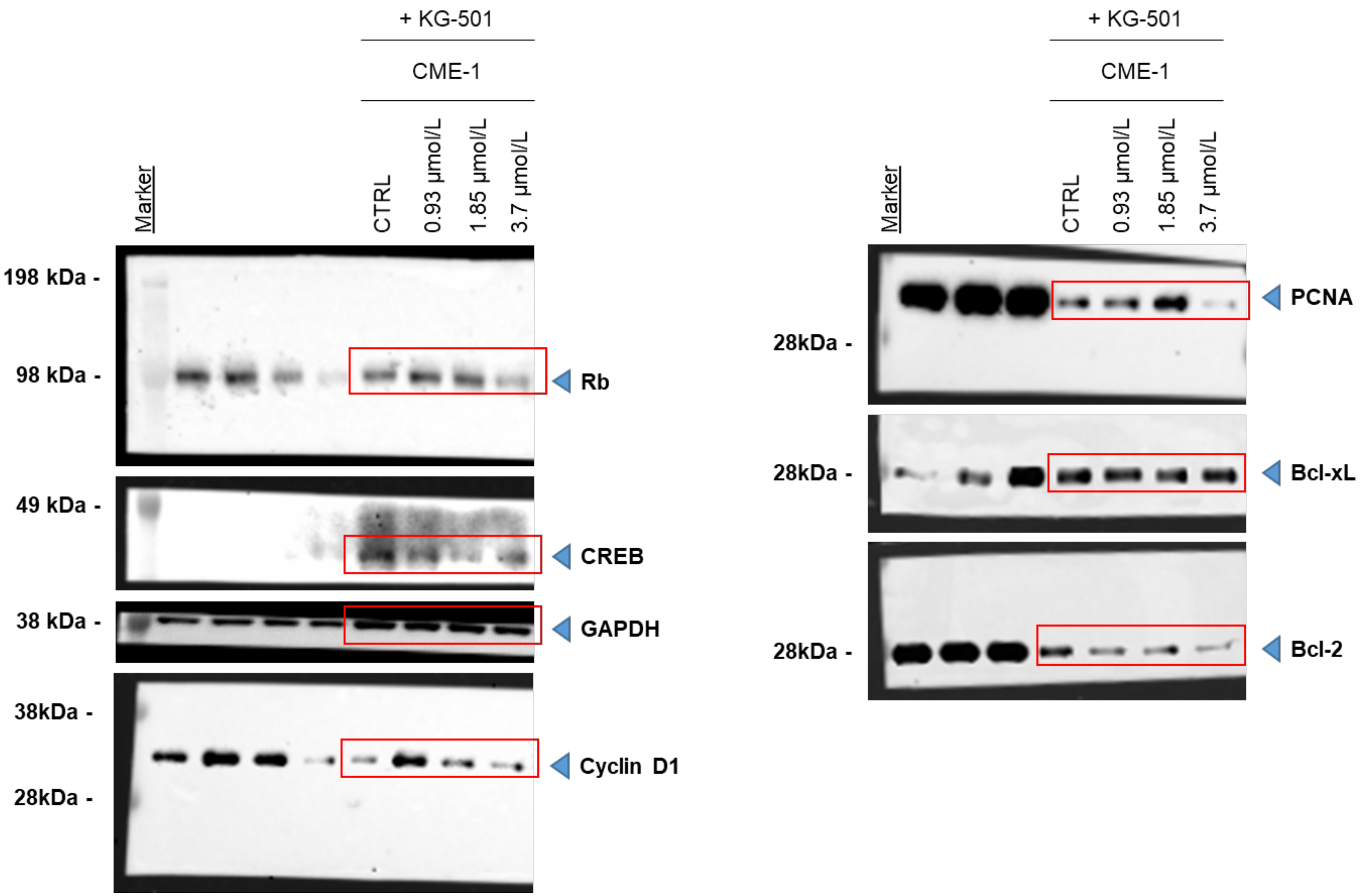

Marker:
SeeBlue Plus2 Pre-Stained Protein Standard
(Thermo Fisher Scientific)
